# Clinical Delays and Comparative Outcomes in Younger and Older Adults with Colorectal Cancer: A Systematic Review

**DOI:** 10.3390/curroncol29110679

**Published:** 2022-11-12

**Authors:** Matthew Castelo, Colin Sue-Chue-Lam, Lawrence Paszat, Adena S. Scheer, Bettina E. Hansen, Teruko Kishibe, Nancy N. Baxter

**Affiliations:** 1 Department of Surgery, University of Toronto, Toronto, ON M5T 1P5, Canada; 2 Institute of Health Policy, Management and Evaluation, Dalla Lana School of Public Health, University of Toronto, Toronto, ON M5T 1P5, Canada; 3 Department of Radiation Oncology, University of Toronto, Toronto, ON M5T 1P5, Canada; 4 Li Ka Shing Knowledge Institute, St Michael’s Hospital, Toronto, ON M5B 1W8, Canada; 5 School of Population and Global Health, University of Melbourne, 207 Bouverie St. Level 5, Melbourne, VIC 3010, Australia

**Keywords:** colorectal neoplasms, young adult, time-to-treatment, delayed diagnosis, systematic review

## Abstract

Outcome disparities between adults <50 with colorectal cancer (CRC) and older adults may be explained by clinical delays. This study synthesized the literature comparing delays and outcomes between younger and older adults with CRC. Databases were searched until December 2021. We included studies published after 1990 reporting delay in adults <50 that made comparisons to older adults. Comparisons were described narratively and stage between age groups was meta-analyzed. 39 studies were included representing 185,710 younger CRC patients and 1,422,062 older patients. Sixteen delay intervals were compared. Fourteen studies (36%) found significantly longer delays among younger adults, and nine (23%) found shorter delays among younger patients. Twelve studies compared time from symptom onset to diagnosis (N younger = 1538). Five showed significantly longer delays for younger adults. Adults <50 years also had higher odds of advanced stage (16 studies, pooled OR for Stage III/IV 1.76, 95% CI 1.52–2.03). Ten studies compared time from diagnosis to treatment (N younger = 171,726) with 4 showing significantly shorter delays for younger adults. All studies showing longer delays for younger adults examined pre-diagnostic intervals. Three studies compared the impact of delay on younger versus older adult. One showed longer delays were associated with advanced stage and worse survival in younger but not older adults. Longer delays among younger adults with CRC occur in pre-diagnostic intervals.

## 1. Introduction

The incidence of colorectal cancer (CRC) in younger adults (<50 years) has increased in a number of countries [1,2,3,4]. Further, CRC mortality among younger adults increased an average of 1.1% annually from 2005 to 2017 [5]. This stands in contrast to older adults, where mortality has decreased by 3% annually and incidence continues to fall [6].

One potential explanation for these disparities may be delays to diagnosis and treatment for younger adults. These delays may be the result of longer time to presentation on the part of the patient, misdiagnosis by physicians, and lack of access to colorectal cancer screening [7]. The US Preventative Services Task Force has recently recommended beginning colorectal cancer screening at age 45 [8], however younger patients are not eligible for screening. The majority of younger adults do not have family histories of CRC, and less than a quarter have a clear predisposing factor such as inflammatory bowel disease [7,9,10]. A previous review has suggested younger adults may take on average 6.2 months to present for initial evaluation after developing symptoms. [7] This evidence base is now dated, with all included studies being over 20 years old, and comparisons to older adults with CRC were not made, providing little insight into the emerging disparity between younger and older adults with CRC. Some authors have explicitly called for a reduction in the time from symptom onset to evaluation in an effort to improve CRC outcomes in younger adults [11], although previous studies have not clearly established a relationship between longer delay and adverse survival in adults <50 years [12,13]. Additionally, after adjustment for patient and disease characteristics, there is evidence younger adults may actually have improved survival compared to older adults [14].

Existing reviews do not examine differences in delay between younger and older patients with CRC [7,15,16], making it difficult to know whether delay might explain the observed divergence in CRC outcomes between these populations. Gaining a better understanding of patterns of delay between younger and older patients will inform efforts to address disparities in care. To address this knowledge gap, we have conducted a systematic review of all observational studies that compared delays between CRC patients <50 years and older adults. Among these studies we also compared the impact of delay on cancer outcomes, CRC stage at presentation, recurrence, and survival between younger and older patients.

## 2. Materials and Methods

We developed a systematic review protocol using the Preferred Reporting Items for Systematic Reviews and Meta-analysis guidelines for protocols (PRISMA-P). [17] Our review was prospectively registered on PROSPERO (Prospective Register of Systematic Reviews—registration number CRD42020179707) and is reported according to the PRISMA guidelines (Supplementary Material Table S1) [18].

### 2.1. Information Sources

We developed a literature search strategy in collaboration with a senior information specialist. We limited the search to observational studies using a published search filter [19]. The electronic databases MEDLINE, Embase, and the Latin American and Caribbean Health Sciences Literature (LILACS) were searched from inception until 2 December 2021 (Supplementary Material Table S2). We limited results to studies published in English, French, Portuguese, and Spanish from 1990 to present. The search strategy was peer reviewed by a second information specialist following the Peer Review of Electronic Search Strategies (PRESS) checklist [20]. We performed a grey literature search according to the Canadian Agency—for Drugs and Technologies in Health Grey Matters checklist [21].

### 2.2. Eligibility Criteria

We included observational studies published after 1990 reporting any delay interval containing time points between symptom onset and treatment start. Studies must have reported delay measures in CRC patients <50 years of age, and additionally made comparisons to older adults with respect to these measures. We excluded studies meeting the following criteria: (i) results not reported for younger adults (age <50 or younger), (ii) majority of patients were pediatric (age <18), (iii) only reported delays to adjuvant therapy (e.g., time between surgery and chemotherapy), (iv) less than 10 younger patients included, (v) conference reports, published abstracts without accompanying complete articles, and (vi) papers that examined delays due to the impact of COVID-19 on health care systems.

### 2.3. Data Management

The DistillerSR (Ottawa, ON, Canada) software platform was used to store retrieved articles and perform the study selection process.

### 2.4. Study Selection

Screening was conducted in three stages. Two reviewers (MC and CS) independently screened titles, titles and abstracts, and finally full texts for results retrieved from the literature search. We resolved conflicts by discussion, with input from a third reviewer (NB) when required.

### 2.5. Data Collection Process

Two reviewers (MC and CS) independently abstracted study information, patient characteristics, tumor characteristics, delay measures, and cancer outcomes stratified by younger and older age. A piloted Microsoft Excel (Washington, DC, USA) data collection sheet was developed to record the data and risk of bias results. We resolved conflicts by discussion, with input from a third reviewer (NB) when required. Authors were contacted for data clarification as needed.

### 2.6. Outcomes and Definitions

The primary outcomes of interest were measures of delay. These included the magnitude and variability of any delay interval falling along the pathway to treatment (Supplementary Material Figure S3). [22] When reported in the studies we also compared stage at presentation, overall survival, disease-free/event-free survival, recurrence, and the impact of delay on these outcomes between younger and older adults. We defined advanced stage as Stage III or IV and early stage as Stage I or II.

### 2.7. Risk of Bias Assessment

Risk of bias was assessed independently by two reviewers (MC and CS). We resolved conflicts by discussion, with input from a third reviewer (NB) when required. The Newcastle-Ottawa Scale [23], and the Aarhus checklist [22] were used to assess the risk of bias for each study. The Aarhus checklist is an international consensus-derived 20-item tool specifically intended to assess the measurement of cancer delay intervals in observational research [22].

### 2.8. Synthesis

Study characteristics and risk of bias results were described narratively. Each study was categorized according to the delay intervals measured. The number of studies and relevant sample sizes of younger and older adults were presented according to each interval. The differences in delay intervals between younger and older adults were described narratively. There was substantial heterogeneity in these reported comparisons, including intervals measured, age cut-offs and categorizations, statistical models, methods of adjustment, and patient populations. Therefore, quantitative synthesis was not possible.

Among studies that reported delays in younger adults, additional comparisons available included stage at presentation, survival, and recurrence, and the impact of delay on these outcomes. Formal meta-analysis was possible for the comparison of advanced stage at presentation between younger and older patients. Pooled odds ratios (ORs) and 95% confidence intervals (CIs) were calculated from weights in a random effects model, treating advanced stage (Stage III/IV) as the outcome of interest. Subgroup analyses were performed between studies that examined colon and rectal cancer together, and each disease site separately. Two sensitivity analyses were performed. The first excluded the largest study, and the second defined advanced stage as only metastatic disease (Stage IV). Heterogeneity was quantified using I^2^ scores and Forest plots. I^2^ scores above 50% were considered high heterogeneity [24]. Similar to the delay comparison, heterogeneity precluded meta-analysis for survival outcomes.

Data were analyzed using R (R Foundation for Statistical Computing, Vienna, Austria), all statistical tests were two-sided, and *p* < 0.05 was considered statistically significant.

## 3. Results

### 3.1. Search Results

**Figure 1:**
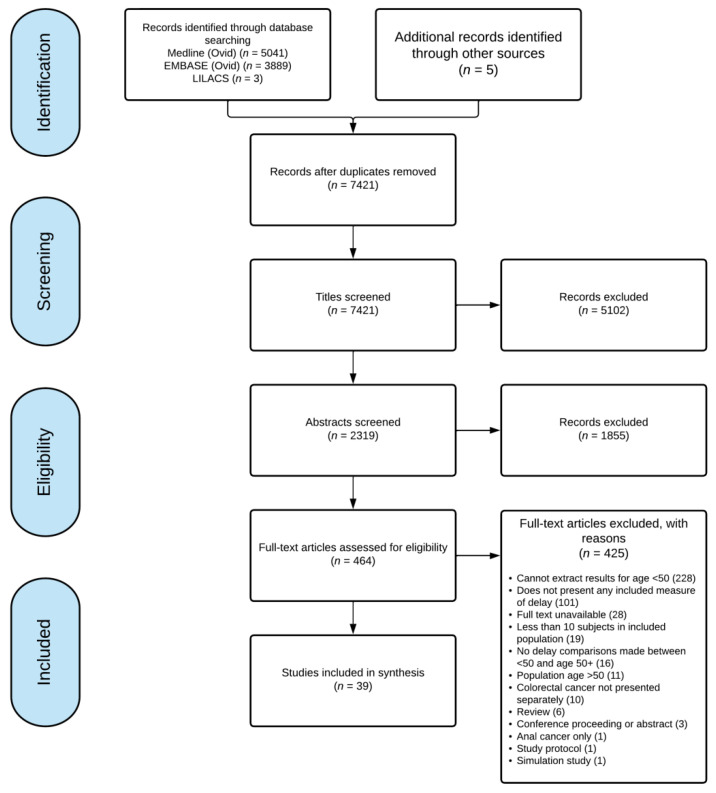
Preferred Reporting Items for Systematic Review and Meta-analysis flow diagram of included studies. Values indicate the number of studies meeting that criteria.

The database and grey literature searches returned 7421 non-duplicated citations (Figure 1). After screening titles and abstracts, we evaluated 464 full texts, and 39 studies [12,13,25,26,27,28,29,30,31,32,33,34,35,36,37,38,39,40,41,42,43,44,45,46,47,48,49,50,51,52,53,54,55,56,57,58,59,60,61] met the inclusion criteria.

### 3.2. Study Characteristics

Table 1 presents the characteristics of 39 studies that reported a delay measure in younger (<50 years) adults with CRC, and made some comparison to delays in older adults. The most recent study was published in 2021, and the oldest in 1992. One study, Lima et al. [61], was published in Portuguese. All other studies were available in English. Three studies reported only an overall sample size without stratifying by younger and older adults (*n* = 14,296). [38,49,50] The remaining studies represented 1,607,772 patients with CRC, of whom 185,710 (11.6%) were classified as younger adults (as per individual study definitions). Most studies defined younger adults as age <50 (28/39; 72%), with the remaining 11 studies using lower age cut-offs (Table 1). Gabriel et al. [34] contributed the overwhelming majority of patients to both the younger and older cohorts, representing 75.5% of the overall sample size. The median number of younger patients among the 39 studies was 87 (IQR 53–565), and the median total sample size was 842 (IQR 308–7031). Seven studies (18%) had less than 50 younger CRC patients.

Studies were performed in sixteen different countries. Canada (8/39; 21%), the United Kingdom (7/39; 18%), and the United States (6/39; 15%) were the most common countries of study origin. The most common data source was primary data collection (22/39; 56%), followed by cancer registries or health administrative data (16/39; 41%). Most studies examined both colon and rectal cancer (32/39; 82%).

### 3.3. Risk of Bias Assessment

The Newcastle-Ottawa Scale [23] scores are presented in Supplementary Material Table S4A. Eighteen studies (46%) did not report on a truly representative cohort, being highly selected single-center studies. Twenty-two studies (56%) did not present comparisons adjusted for any potential confounders. Studies generally drew younger and older adults from the same community, and used secure records such as medical records to determine age. Twelve studies (31%) included statements or figures demonstrating loss to follow-up. The Aarhus Checklist was used to assess risk of bias specific to delay studies (Supplementary Material Table S4B) [22]. Of note, seven studies (18%) were published prior to the Aarhus Checklist’s availability. Studies reliably identified the beginning and end points of delay intervals, but few provided precise and repeatable definitions of how these time points were calculated. For studies that included the date of symptom onset, most (8/15; 53%) discussed the potential biases when measuring this time point. Eight studies (21%) explicitly referred to established definitions for delay intervals and referenced theoretical frameworks. Seven studies included data from questionnaires or patient interviews. Of these, five discussed the potential biases resulting from how and when the question is being asked, and the difficulty in determining patient-reported time points.

### 3.4. Delay Measures between Younger and Older Patients

**Figure 2:**
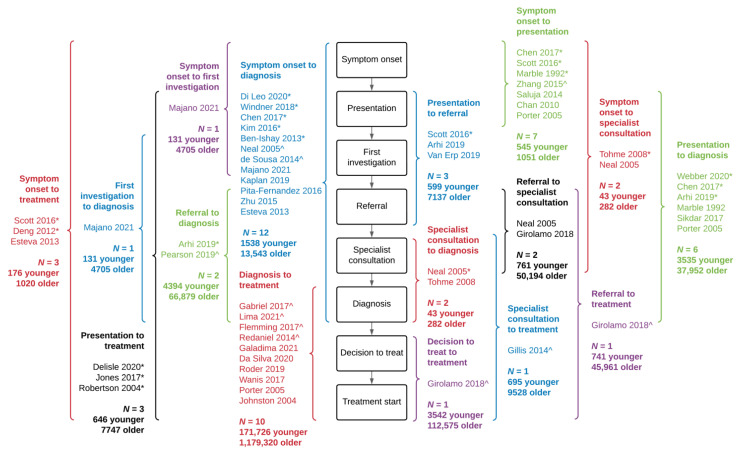
Reported comparisons of delay intervals between younger and older adults with colorectal cancer, grouped by interval on the pathway to treatment. Shown is the number of studies in each interval, and the sample size of younger and older adults. Studies that did not report sample sizes for both younger and older are not included in the number of patients. *Indicates study found significantly longer delay among younger adults with colorectal cancer and ^indicates study found significantly shorter delay among younger adults.

While all studies made some comparison between delay in younger and older adults with CRC, there was substantial heterogeneity in reporting. Measures used to compare delay included medians, means, and proportions of patients reaching a delay cut-off. These comparisons were also heterogeneously reported, including odds ratios, risk ratios, differences in means/medians/90th percentiles, and additional days of delay. Sixteen unique delay intervals were compared among the 39 studies (Figure 2). Five interval measures were evaluated in only one study [13,42,60].

### 3.5. Interval from Symptom Onset to Diagnosis

Time between symptom onset and diagnosis was reported by the largest number of studies (*n* = 12), comparing 1538 younger adults to 13,543 older adults. Five of these studies found younger adults had significantly longer times between symptom onset and diagnosis, two found shorter delays for younger adults, and the remaining five showed no significant differences (Figure 2 and Supplementary Material Table S5). Zhu et al. [29], Kim et al. [12], and Majano et al. [60] were the largest studies reporting sample sizes of younger patients for time between symptom onset and diagnosis. Zhu et al. [29] compared 83 CRC patients under 30 to 4911 older patients with a mean age of 58.9 years, showing no significant difference in time from symptom onset to diagnosis (younger mean 4.6 months vs. older mean 6.2 months, *p* = 0.691). Kim et al. [12] reported significantly longer delays among 693 patients ≤45 years compared to patients aged 56–65 (younger mean 52.9 days vs. older mean 33.3 days, *p* = 0.001). Both comparisons were unadjusted. In an adjusted analysis of 131 CRC patients under 45 compared to 4705 older patients, Majano et al. [60] showed a non-significant difference in median time from symptom onset to diagnosis for rectal cancer (age <45 additional 59.0 days, 95% CI −8.5 to 126.5 vs. age 55–64) and colon cancer (age <45 additional 82.0 days, 95% CI −24.5 to 188.5 vs. age 55–64).

### 3.6. Advanced Stage at Presentation for Younger and Older Patients

**Figure 3:**
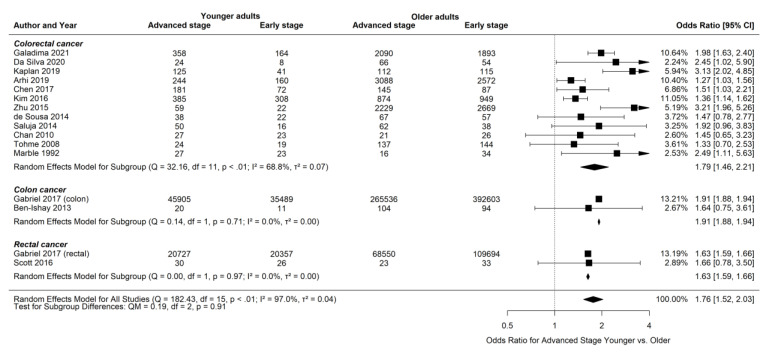
Random effects meta-analysis of advanced stage at diagnosis by age category. Subgroup analyses were performed by type of cancer studied. Advanced stage was defined as Stage III/IV and early stage was defined as Stage I/II. Squares indicate individual study effect sizes with arrows indicating confidence intervals. Black diamonds indicate pooled effect sizes and confidence intervals [12,29,31,32,36,40,43,47,48,53,54,57].

Sixteen studies [12,25,29,31,32,34,36,38,40,43,44,47,48,53,54,57] compared stage at presentation between younger and older patients with CRC. Ten studies found significantly higher odds of advanced stage (Stage III or IV) among younger patients, and the remaining studies found no significant difference (Table 2 and Supplementary Material Table S6). None of the studies found lower odds of advanced stage among younger patients. A random-effects meta-analysis was performed, with very high heterogeneity (I^2^ = 97.0%; Figure 3). Younger age was associated with significantly higher odds of advanced stage at presentation (pooled OR 1.76, 95% CI 1.52–2.03). There was no significant subgroup difference by cancer site (colorectal vs. colon only vs. rectal only; *p* = 0.91; Figure 3). A sensitivity analysis excluding the large Gabriel et al. [34] study came to similar conclusions (Supplementary Material Figure S7), as did a sensitivity analysis examining metastatic disease versus non-metastatic disease (Supplementary Material Figure S8).

### 3.7. Interval from to Diagnosis to Treatment Start

Due to the study by Gabriel et al. [34], the largest comparison by sample size was time between diagnosis and treatment (Figure 2). Using National Cancer Database (NCDB) data, Gabriel et al. [34] reported 155,090 younger adults experienced a mean delay of 11.18 days for colon cancer compared to a mean delay of 13.18 days in 1,058,102 older adults. This difference was statistically significant (*p* < 0.001), but of questionable clinical relevance. The reported mean difference for rectal cancer patients was smaller (age <50 mean 22.02 days vs. age 60+ mean 22.48 days, *p* < 0.001). [34] Three additional studies reported this interval also found significantly shorter delays for younger adults [28,41,61]. No study found longer intervals between diagnosis and treatment for younger adults.

### 3.8. Remaining Delay Intervals

Three studies [25,45,46] examined the entire interval from symptom onset to treatment start, two of which showed longer delays for younger adults. Scott et al. [25] reported significantly longer delays among younger adults (younger median 217 days [*n* = 56] vs. older median 58 days [*n* = 56], *p* < 0.0001). Deng et al. [46] found mean time from symptoms onset to treatment was 120.3 days for younger adults compared to 74.4 days in older adults with colon cancer (*p* = 0.035). The same comparison was not significantly different for rectal cancer patients. [46] Esteva et al. [45] reported no significant difference in time between symptom onset and treatment for 45 younger adults compared to older adults in five regions in Spain (age <50 median 149.0 days vs. age 50–64 median 133.0 days, *p* = 0.20).

The interval from presentation to treatment was compared in three studies, [37,52,55] with all three finding significantly longer delays for younger adults (Figure 2). When studies were categorized by finding irrespective of interval studied, 14 studies (36%) found significantly longer delays among younger adults, nine (23%) found shorter delays among younger patients, two (5%) showed mixed findings, and the remaining 14 studies (36%) found no significant differences (Table 2). All studies that found longer delays among younger adults included pre-diagnostic time.

### 3.9. Impact of Delay on Outcomes between Younger and Older Patients

Three studies [12,13,36] evaluated the impact of delay on either stage at presentation or survival, stratified by younger and older adults. Table 3 presents the results for each study. Kim et al. [12] was the only study showing increased odds of advanced stage with longer delay for younger adults (symptom onset to diagnosis >3 months OR of Stage III/IV 6.33, 95% CI 3.05–13.12). In the same study, longer delay was not associated with advanced stage in older adults. Conversely, Chen et al. [36] found advanced stage was associated with a shorter interval from symptom onset to diagnosis for both younger and older adults. Girolamo et al. [13] similarly found lower odds of advanced stage with longer delays in older adults, and no significant association for younger adults.

Kim et al. [12] also reported stratified associations between delay and survival. In younger adults, longer delay was associated with worse survival only in the adjusted Cox model (symptom onset to diagnosis >3 months adjusted HR 2.57, 95% CI 1.34–4.94). Longer delay was not associated with worse survival in older adults. Girolamo et al. [13] reported higher 1-year survival among those with longer delays for both younger and older adults, although p-values were not provided.

### 3.10. Survival and Recurrence for Younger and Older Patients

Fifteen studies [12,13,25,26,28,32,34,40,41,43,44,48,53,54,55] compared survival between younger and older colorectal cancer patients. There was heterogeneity among outcomes (cancer-specific survival, overall survival, 30-day mortality), adjustment for covariates, follow-up time, and analysis strategy (time-to-event models versus binomial models). Formal meta-analysis was not possible. Two studies [12,53] (13%) found significantly worse survival among younger patients, 7 (47%) studies found worse survival in only unadjusted analyses or non-significant differences, and the remaining 6 studies (40%) showed significantly better survival among younger patients (Table 2 and Supplementary Material Table S9).

Five studies [12,32,43,48,54] (13%) examined disease recurrence or event-free/cancer-free survival (Table 2 and Supplementary Material Table S9). Two studies [12,32] showed worse outcomes for younger patients, and the remaining three studies [43,48,54] showed no significant differences.

## 4. Discussion

In this systematic review and meta-analysis of 39 studies comparing delay measures between adults <50 and older adults, we found a highly heterogeneous body of literature that examined sixteen distinct delay intervals and various patient populations, using differing age cut-offs, methods of adjustment, and sample sizes. However, these studies indicate that when younger adults do experience longer delays, the delay generally occurs before diagnosis. Indeed, studies finding longer delays in younger adults all compared intervals including pre-diagnostic time. However, the included studies reached contradictory conclusions regarding whether these pre-diagnostic delays lead to worse outcomes in younger adults compared to older adults. We found that younger patients were at higher risk of presenting with advanced stage at presentation (Stage III/IV); however there was no consistent relationship between survival and age at presentation.

Evaluating delay intervals in cancer research is complex, and numerous biases have been described when assessing time points along the pathway to treatment. [22] We were unable to perform a meta-analysis for delay interval comparisons between younger and older patients for reasons of heterogeneity. However, we are able to generalize based on the literature reviewed. Specifically, we found no evidence that younger adults experience longer delays from diagnosis to treatment. In fact, all four studies that found significant differences for this interval showed younger adults had shorter times from diagnosis to treatment. This interval tends to be widely reported in the literature, as health administrative databases and cancer registries are well positioned to measure cancer diagnosis dates and treatment dates [26,28,30,34,41,49,51,57,61,62,63].

Examining pre-diagnostic intervals are more challenging, especially date of symptom onset [22]. Health administrative databases are largely unsuitable for determining this date and patient questionnaires or detailed chart review must be used. Patient-reported dates may be subject to recall bias, and the quality of documentation in charts is variable [22]. Unsurprisingly, the sample sizes of comparisons using these time points were generally small. However, we did find several pre-diagnostic intervals with larger studies making comparisons to older adults. In 12 studies reporting a total of more than 1538 younger patients, five [12,33,36,44,56] found significantly longer times from symptom onset to diagnosis compared to older patients. Contained within this interval, two studies [27,31] reported time from referral to diagnosis for over 4000 younger patients and came to conflicting conclusions with regard to length of delay for younger versus older patients. Taken together, the interval between symptom onset and presentation (so-called patient delay) appears to be an important potential source of disparity between younger and older patients.

Younger patients may be at higher risk of experiencing delays from symptom onset to presentation [7,11,25] as they are not eligible for screening, and many initial symptoms of colorectal cancer are nonspecific or have easily offered alternative diagnoses such as hemorrhoidal disease. Experts have suggested patient education about the increasing risk of colorectal cancer among young people, increased awareness of alarm symptoms, and destigmatizing colorectal cancer as options to improve early detection [11]. However, the evidence base examining time from symptom onset to presentation is very weak. In our review, seven studies compared this interval between 545 younger and 1051 older adults, and only three demonstrated that younger patients have longer delays [25,36,53]. An additional study reported time from symptom onset to first investigation, with no significant difference between the age groups [60]. Interventions targeting the pre-diagnostic period are more attractive given these results, although there is a lack of research in this area, especially among younger adults. Research is needed to identify modifiable patient, clinician, and system factors that may be amenable to intervention.

However, the observed relationship in retrospective studies between delay and adverse outcomes in CRC is itself uncertain [15,16]. Large, methodologically robust studies of mostly older adults have come to differing conclusions [28,64,65,66]. While some have described worse survival for cancer patients with longer delays [65,67], few studies examine this association in younger adults with CRC [12,13]. Some have suggested fast-growing tumors may cause troublesome symptoms sooner and lead to shorter delay intervals [64]. These cancers also tend to be more aggressive and result in worse outcomes. Similarly, there is also not a clear relationship between increased delay and stage [15]. We were able to identify three studies that compared the impact of delays on stage at presentation or survival between younger and older adults [12,13,36]. Kim et al. [12] was the only study to show delay was associated with more advanced stage and worse survival in younger adults compared to older adults.

Although not the primary outcome of this review, we have shown younger patients present with more advanced colorectal cancer (pooled OR 1.76, 95% CI 1.52–2.03; I^2^ = 97.0%), and may have improved survival compared to older patients once adjusted for stage [14]. These comparisons were limited by our inclusion criteria of studies that reported delay measures among young adults, and high heterogeneity. There are several hypotheses regarding why younger patients present with more advanced disease, including adverse histology, intrinsic biological aggressiveness, and tumor location [68].

Strengths of this review include its size and scope—we endeavored to identify a global literature, including four languages in our search criteria. We categorized delay comparisons according to international consensus-derived definitions, referenced established theoretical frameworks for delay, and utilized a formal cancer delay quality tool (the Aarhus Checklist) [22,69]. Previous systematic reviews examining the relationships between delay, stage, and survival have not considered younger adults as a distinct population, and did not systematically classify delay intervals as we have done [15,16].

There are limitations to this review. There are important factors that influence delay that we were unable to incorporate into our analysis, including health care system differences, screening eligibility, co-morbidity, intrinsic biologic differences, and non-linear relationships with age. This was due to sparse reporting and the large number of distinct delay interval comparisons. The very large American study from Gabriel et al. [34] contributed a majority of patients from both the younger and older group, and may be overrepresented in our conclusions. We did not include studies reporting on care provided during the COVID-19 pandemic, and our results may not be applicable to delays experienced in this context. As discussed, comparisons of pre-diagnostic intervals include few younger adults and conclusions with respect to these intervals are likely limited by low power. Finally, all the studies included in this review were observational studies, many of which were single-centre studies of selected patient groups. These studies are subject to the biases of retrospective cohort studies, including selection bias and unmeasured confounding [70]. Despite these limitations, this review is the most comprehensive assessment of delays in younger versus older adults with CRC available [7].

While a large number of studies have examined the topic, our understanding of delay and its clinical impacts in younger adults with CRC remains incomplete. It appears younger patients may have longer pre-diagnostic delays but experience shorter delays from diagnosis to treatment compared to older adults. High-quality studies are needed to confirm these findings and, critically, examine the potential for reducing delays to improve survival or stage at presentation. Attempts to reduce clinical delays among younger adults with CRC should focus on the pre-diagnostic time period.

## Figures and Tables

**Table 1:** Characteristics of included studies (*n* = 39) [12,13,25,26,27,28,29,30,31,32,33,34,35,36,37,38,39,40,41,42,43,44,45,46,47,48,49,50,51,52,53,54,55,56,57,58,59,60,61].

Study	Characteristic							
	Definition of Young	No. Younger	No. Older	Country	Study Type	Data Source	Years of Study	Number of Sites
Colon and Rectal Cancer								
Lima 2021 [61]	<50	14,675	64,472	Brazil	Retrospective cohort	Cancer registry/health administrative data	2006–2015	Population-based
Majano 2021 [60]	<45	131	4705	UK	Retrospective cohort	Cancer registry/health administrative data	2011–2015	Population-based
Galadima 2021 [57]	<50	522	3983	USA	Retrospective cohort	Cancer registry/health administrative data	2008–2016	Population-based
Delisle 2020 [55]	<50	519	6417	Canada	Retrospective cohort	Cancer registry/health administrative data	2004–2014	Population-based
Di Leo 2020 [56]	<50	54	494	Italy	Retrospective cohort	Primary data collection	2015–2018	1
Da Silva 2020 [54]	<50	39	145	Brazil	Retrospective cohort	Primary data collection	2013–2018	1
Webber 2020 [59]	<50	1902	22,059	Canada	Retrospective cohort	Cancer registry/health administrative data	2008–2012	Population-based
Van Erp 2019 [58]	<50	35	274	Netherlands	Retrospective cohort	Cancer registry/health administrative data	2007–2011	Population-based
Roder 2019 [30]	<50	91	1584	Australia	Retrospective cohort	Cancer registry/health administrative data	2000–2010	4
Arhi 2019 [31]	<50	508	6807	UK	Retrospective cohort	Cancer registry/health administrative data	2006–2013	Population-based
Kaplan 2019 [32]	20–25	141	237	Turkey	Retrospective cohort	Primary data collection	2003–2015	20
Pearson 2019 [27]	<50	3886	60,072	UK	Retrospective cohort	Cancer registry/health administrative data	2014–2015	Population-based
Windner 2018 [33]	<50	41	55	New Zealand	Survey study	Primary data collection	-	-
Girolamo 2018 [13]	15–44	3542	112,575	UK	Retrospective cohort	Cancer registry/health administrative data	2009–2013	Population-based
Gabriel 2017 [34]	<50	155,090	1,058,102	USA	Retrospective cohort	Cancer registry/health administrative data	1998–2011	Population-based
Sikdar 2017 [35]	<50	822	8804	Canada	Retrospective cohort	Cancer registry/health administrative data	2004–2010	Population-based
Chen 2017 [36]	<50	253	232	USA	Retrospective cohort	Primary data collection	2008–2014	1
Kim 2016 [12]	≤45	693	1823	Republic of Korea	Retrospective cohort	Primary data collection	2006–2011	1
Pita-Fernandez2016 [38]	<50	-	942 younger and older	Spain	Retrospective cohort	Primary data collection	1994–2000	1
Zhu 2015 [29]	<30	83	4911	China	Retrospective cohort	Primary data collection	1995–2013	1
Saluja 2014 [40]	<40	66	100	India	Retrospective cohort	Primary data collection	2003–2012	1
Redaniel 2014 [41]	15–44	921	45,590	UK	Retrospective cohort	Cancer registry/health administrative data	1996–2009	Population-based
de Sousa 2014 [43]	<50	66	149	Brazil	Retrospective cohort	Primary data collection	2006–2010	1
Esteva 2013 [45]	<50	45	732	Spain	Cross-sectional study	Primary data collection	2006–2008	5 regions in Spain
Deng 2012 [46]	<50	75	232	China	Prospective cohort	Primary data collection	2008–2009	1
Chan 2010 [47]	<40	53	47	Sri Lanka	Retrospective cohort	Primary data collection	1996–2008	1
Tohme 2008 [48]	<45	43	282	Lebanon	Retrospective cohort	Primary data collection	1995–2005	1
Porter 2005 [49]	<50	-	110 younger and older	Canada	Prospective cohort	Primary data collection	2001	1
Neal 2005 [50]	<45	-	13,244 younger and older	UK	Survey study	Primary data collection	2002	Population-based
Johnston 2004 [51]	25-50	95	503	Canada	Retrospective cohort	Cancer registry/health administrative data	1992–2000	Population-based
Robertson 2004 [52]	<50	53	1018	UK	Retrospective cohort	Cancer registry/health administrative data	1997–1998	Population-based
Marble 1992 [53]	<40	50	50	USA	Retrospective cohort	Primary data collection	1935–1988	1
Colon cancer								
Flemming 2017 [28]	<50	246	4080	Canada	Retrospective cohort	Cancer registry/health administrative data and primary data collection	2002–2008	Population-based
Wanis 2017 [26]	<50	47	861	Canada	Retrospective cohort	Primary data collection	2006–2015	1
Jones 2017 [37]	<50	74	312	USA	Prospective cohort	Primary data collection	2010–2013	9
Gillis 2014 [42]	<50	695	9528	Canada	Prospective cohort	Cancer registry/health administrative data	2002–2008	Population-based
Ben-Ishay 2013 [44]	<50	31	205	Israel	Retrospective cohort	Primary data collection	2000–2009	1
Rectal cancer								
Scott 2016 [25]	<50	56	56	USA	Case control	Primary data collection	1997–2007	1
Zhang 2015 [39]	<50	67	566	China	Prospective cohort	Primary data collection	2008–2009	1

**Table 2:** Comparison of outcomes between older and younger colorectal cancer patients. Shown are delay measures (all intervals), stage at presentation, overall survival, and disease-free survival/recurrence.

Finding	Studies
Delay measures	
Longer delays among younger patients (*N* = 14)	Webber 2020, Delisle 2020, Di Leo 2020, Windner 2018, Chen 2017, Jones 2017, Kim 2016, Scott 2016, Ben-Ishay 2013, Robertson 2004, Arhi 2019, Deng 2012, Tohme 2008, Marble 1992
No significant differences (*N* = 14)	Majano 2021, Galadima 2021, Da Silva 2020, Van Erp 2019, Roder 2019, Kaplan 2019, Wanis 2017, Pita-Fernandez 2016, Zhu 2015, Saluja 2014, Esteva 2013, Chan 2010, Porter 2005, Johnston 2004
Mixed findings (*N* = 2)	Neal 2005, Sikdar 2017
Shorter delays among younger patients (*N* = 9)	Girolamo 2018, Gabriel 2017, Lima 2021, Pearson 2019, Flemming 2017, Zhang 2015, Redaniel 2014, Gillis 2014, de Sousa 2014
Stage at presentation	
Worse stage at presentation among younger patients (*N* = 10)	Galadima 2021, Da Silva 2020, Arhi 2019, Kaplan 2019, Gabriel 2017, Chen 2017, Kim 2016, Zhu 2015, Marble 1992, Pita-Fernandez 2016
No significant difference (*N* = 7)	Pita-Fernandez 2016, Scott 2016, Saluja 2014, de Sousa 2014, Ben-Ishay 2013, Chan 2010, Tohme 2008
Survival	
Worse survival among younger patients (*N* = 3)	Kim 2016, Marble 1992, Kaplan 2019
No significant difference (*N* = 7)	Da Silva 2020, Kaplan 2019, Scott 2016, Saluja 2014, de Sousa 2014, Ben-Ishay 2013, Tohme 2008
Better survival among younger patients (*N* = 6)	Delisle 2020, Girolamo 2018, Gabriel 2017, Flemming 2017, Wanis 2017, Redaniel 2014
Recurrence and disease-free survival	
Worse recurrence among younger patients (*N* = 2)	Kaplan 2019, Kim 2016
No significant difference (*N* = 3)	Da Silva 2020, de Sousa 2014, Tohme 2008

**Table 3:** Associations between delay intervals and stage at presentation and survival between younger and older adults with colorectal cancer.

Study	Outcome	Finding in Younger Adults *	Finding in Older Adults *
**Stage**			
Kim 2016 [12]	Advanced stage (Stage III/IV)	*Longer delay associated with higher odds of advanced stage* Symptom onset to diagnosis <1 month: Reference 1–3 months: OR 3.01 (1.77–5.12) >3 months: OR 6.33 (3.05–13.12)	*Delay not associated with advanced stage* Symptom onset to diagnosis <1 month: Reference 1–3 months: OR 1.28 (0.81–2.02) >3 months: OR 1.46 (0.81–2.62)
Chen 2017 [36]	Advanced stage (Stage III/IV)	*Advanced stage associated with shorter delays* Symptom onset to presentation Stage III/IV vs. Stage I/II: median difference—30 days Presentation to diagnosis Stage III/IV vs. Stage I/II: median difference —0 days Symptom onset to diagnosis Stage III/IV vs. Stage I/II: median difference—40 days	*Mixed findings* Symptom onset to presentation Stage III/IV vs. Stage I/II: median difference +9 days Presentation to diagnosis Stage III/IV vs. Stage I/II: median difference—14 days Symptom onset to diagnosis Stage III/IV vs. Stage I/II: median difference—5 days
Girolamo 2018 [13] **	Advanced stage (Stage III/IV)	*Delay not associated with advanced stage* Referral to specialist consultation >2 weeks: OR 1.43 (0.65–3.52) Decision to treat to treatment >31 days: OR 0.76 (0.43–1.39) Referral to treatment >62 days: OR 1.03 (0.68–1.57)	*Longed delay associated with lower odds of advanced stage for 2 intervals* Referral to specialist consultation >2 weeks: OR 0.84 (0.76–0.92) Decision to treat to treatment >31 days: OR 0.66 (0.61–0.72) Referral to treatment >62 days: OR 0.99 (0.95–1.04)
**Survival**			
Kim 2016 [12]	Overall survival	*Longer delay associated with worse survival in adjusted analysis only* Symptom onset to diagnosis, unadjusted <1 month: Reference 1–3 months: HR 1.41 (0.86–2.31) >3 months: HR 1.69 (0.99–2.91) Symptom onset to diagnosis, adjusted for sex and tumor differentiation <1 month: Reference1–3 months: HR 1.62 (0.95–2.76) >3 months: HR 2.57 (1.34–4.94)	*Delay not associated with survival* Symptom onset to diagnosis, unadjusted <1 month: Reference 1–3 months: HR 1.05 (0.59–1.87) >3 months: HR 0.75 (0.41–1.35)
Girolamo 2018 [13]	1-year overall survival	*Longer delay associated with improved survival* Referral to specialist consultation <2 weeks vs. >2 weeks 88.9% (86.6–91.2) vs. 90.1% (80.9–99.3) Decision to treat to treatment <31 days vs. >31 days 89.8% (88.8–90.8) vs. 94.8% (89.1–100.0) Referral to treatment <62 days vs. >62 days 90.7% (88.3–93.0) vs. 94.0% (90.1–97.9)	*Longer delay associated with improved survival* Referral to specialist consultation <2 weeks vs. >2 weeks Age 45–54: 89.0% (87.9–90.1) vs. 89.4% (84.9–93.9) Age 55–64: 86.0% (85.3–86.8) vs. 88.5% (85.5–91.5) Age 65–74: 83.1% (82.4–83.7) vs. 86.5% (83.9–89.2) Age 75+: 76.9% (76.3–77.6) vs. 79.7% (76.9–82.4) Decision to treat to treatment <31 days vs. >31 days Age 45–54: 89.0% (87.9–90.1) vs. 91.0% (90.4–91.6) Age 55–64: 86.0% (85.3–86.8) vs. 91.9% (91.6–92.3) Age 65–74: 83.1% (82.4–83.7) vs. 91.0% (90.7–91.3) Age 75+: 76.9% (76.3–77.6) vs. 85.7% (85.3–86.1) Referral to treatment <62 days vs. >62 days Age 45–54: 91.6% (90.5–92.7) vs. 93.9% (92.1–95.8) Age 55–64: 90.3% (89.6–91.1) vs. 91.5% (90.2–92.8) Age 65–74: 89.4% (88.8–90.1) vs. 89.7% (88.7–90.8) Age 75+: 85.4% (84.7–86.1) vs. 90.9% (89.9–91.8)

* Presented are effect estimates and 95% confidence intervals. Analyses are unadjusted unless otherwise specified. ** Values for older age groups were combined to calculate a single effect for older adults; OR—odds ratio, HR—hazard ratio.

## Supplementary Materials

The following supporting information can be downloaded at: https://www.mdpi.com/article/10.3390/curroncol29110679/s1, Table S1: PRISMA checklist; Table S2: Search strategy for Medline; Figure S3: The pathway to treatment; Table S4A: Scoring for the risk of bias tools including the Newcastle-Ottawa Scale for Cohort Studies; Table S4B: Scoring for the risk of bias tools including the Aarhus checklist; Table S5: Detailed comparison of delay measures between younger and older adults with colorectal cancer; Table S6: Advanced stage at diagnosis comparing older and younger patients; Figure S7: Random effects meta-analysis of advanced stage at diagnosis by age category sensitivity analysis excluding Gabriel et al. study; Figure S8: Random effects meta-analysis of advanced stage at diagnosis by age category sensitivity analysis of metastatic disease; Table S9: Survival and recurrence outcomes among younger and older patients.
