# Supplementary material for: Clinical Delays and Comparative Outcomes in Younger and Older Adults with Colorectal Cancer: A Systematic Review
Source: Curr Oncol. 2022 Nov 12;29(11):8609–25. doi: 10.3390/curroncol29110679 (PMC9689013; doi:10.3390/curroncol29110679)
Supplement: Supplementary file 1 [file curroncol-29-00679-s001.zip › curroncol-1966741-supplementary.pdf]

Supplementary Material

Supplementary Material Table S1. PRISMA checklist.[18]

Section and Topic	Item #	Checklist item	Location where item is reported
**TITLE**			
Title	1	Identify the report as a systematic review.	1
**ABSTRACT**			
Abstract	2	See the PRISMA 2020 for Abstracts checklist.	2
**INTRODUCTION**			
Rationale	3	Describe the rationale for the review in the context of existing knowledge.	4
Objectives	4	Provide an explicit statement of the objective(s) or question(s) the review addresses.	4-5
**METHODS**			
Eligibility criteria	5	Specify the inclusion and exclusion criteria for the review and how studies were grouped for the syntheses.	5-6
Information sources	6	Specify all databases, registers, websites, organisations, reference lists and other sources searched or consulted to identify studies. Specify the date when each source was last searched or consulted.	5-6
Search strategy	7	Present the full search strategies for all databases, registers and websites, including any filters and limits used.	Supplementary Material 2
Selection process	8	Specify the methods used to decide whether a study met the inclusion criteria of the review, including how many reviewers screened each record and each report retrieved, whether they worked independently, and if applicable, details of automation tools used in the process.	6
Data collection process	9	Specify the methods used to collect data from reports, including how many reviewers collected data from each report, whether they worked independently, any processes for obtaining or confirming data from study investigators, and if applicable, details of automation tools used in the process.	6
Data items	10a	List and define all outcomes for which data were sought. Specify whether all results that were compatible with each outcome domain in each study were sought (e.g. for all measures, time points, analyses), and if not, the methods used to decide which results to collect.	6-7
	10b	List and define all other variables for which data were sought (e.g. participant and intervention characteristics, funding sources). Describe any assumptions made about any missing or unclear information.	6-7
Study risk of bias assessment	11	Specify the methods used to assess risk of bias in the included studies, including details of the tool(s) used, how many reviewers assessed each study and whether they worked independently, and if applicable, details of automation tools used in the process.	7
Effect measures	12	Specify for each outcome the effect measure(s) (e.g. risk ratio, mean difference) used in the synthesis or presentation of results.	7-8
Synthesis methods	13a	Describe the processes used to decide which studies were eligible for each synthesis (e.g. tabulating the study intervention characteristics and comparing against the planned groups for each synthesis (item #5)).	7-8
	13b	Describe any methods required to prepare the data for presentation or synthesis, such as handling of missing summary statistics, or data conversions.	7-8
	13c	Describe any methods used to tabulate or visually display results of individual studies and syntheses.	7-8
	13d	Describe any methods used to synthesize results and provide a rationale for the choice(s). If meta-analysis was performed, describe the model(s), method(s) to identify the presence and extent of statistical heterogeneity, and software package(s) used.	7-8
	13e	Describe any methods used to explore possible causes of heterogeneity among study results (e.g. subgroup analysis, meta-regression).	7-8
	13f	Describe any sensitivity analyses conducted to assess robustness of the synthesized results.	7-8
Reporting bias assessment	14	Describe any methods used to assess risk of bias due to missing results in a synthesis (arising from reporting biases).	7-8
Certainty assessment	15	Describe any methods used to assess certainty (or confidence) in the body of evidence for an outcome.	7-8
**RESULTS**			
Study selection	16a	Describe the results of the search and selection process, from the number of records identified in the search to the number of studies included in the review, ideally using a flow diagram.	8, Figure 1
	16b	Cite studies that might appear to meet the inclusion criteria, but which were excluded, and explain why they were excluded.	Figure 1
Study characteristics	17	Cite each included study and present its characteristics.	8, Table 1
Risk of bias in studies	18	Present assessments of risk of bias for each included study.	9, Supplementary Material 4
Results of individual studies	19	For all outcomes, present, for each study: (a) summary statistics for each group (where appropriate) and (b) an effect estimate and its precision (e.g. confidence/credible interval), ideally using structured tables or plots.	Figure 2, Table 2, Table 3, Figure 3
Results of syntheses	20a	For each synthesis, briefly summarise the characteristics and risk of bias among contributing studies.	8-13
	20b	Present results of all statistical syntheses conducted. If meta-analysis was done, present for each the summary estimate and its precision (e.g. confidence/credible interval) and measures of statistical heterogeneity. If comparing groups, describe the direction of the effect.	8-13
	20c	Present results of all investigations of possible causes of heterogeneity among study results.	8-13
	20d	Present results of all sensitivity analyses conducted to assess the robustness of the synthesized results.	8-13
Reporting biases	21	Present assessments of risk of bias due to missing results (arising from reporting biases) for each synthesis assessed.	8-13

Section and Topic	Item #	Checklist item	Location where item is reported
Certainty of evidence	22	Present assessments of certainty (or confidence) in the body of evidence for each outcome assessed.	8-13
**DISCUSSION**			
Discussion	23a	Provide a general interpretation of the results in the context of other evidence.	13-14
	23b	Discuss any limitations of the evidence included in the review.	17
	23c	Discuss any limitations of the review processes used.	17
	23d	Discuss implications of the results for practice, policy, and future research.	13-17
**OTHER INFORMATION**			
Registration and protocol	24a	Provide registration information for the review, including register name and registration number, or state that the review was not registered.	5
	24b	Indicate where the review protocol can be accessed, or state that a protocol was not prepared.	5
	24c	Describe and explain any amendments to information provided at registration or in the protocol.	5
Support	25	Describe sources of financial or non-financial support for the review, and the role of the funders or sponsors in the review.	1
Competing interests	26	Declare any competing interests of review authors.	1
Availability of data, code and other materials	27	Report which of the following are publicly available and where they can be found: template data collection forms; data extracted from included studies; data used for all analyses; analytic code; any other materials used in the review.	1

Supplementary Material Table S2. Search strategy for Medline (original search; updated December 2, 2021)

Medline

1 exp Colorectal Neoplasms/ (197733)
2 exp Intestine, Large/ and (Neoplasms/ or Carcinoma/ or Adenocarcinoma/ or Neoplasm Metastasis/) (8771)
3 ((neoplas* or cancer* or carcinom* or adenocarcinoma* or tumo?r* or malignan* or metastas?s) adj7 (colorect* or colon or colons or colonic or colonoscop* or rect* or sigmoid)).tw,kf. (234590)
4 1 or 2 or 3 (280596)
5 Delayed Diagnosis/ (6092)
6 Time-to-Treatment/ (6144)
7 Time Factors/ (1177228)
8 (delay* adj10 (presentation* or referral* or diagnos* or colonoscop* or surg* or treatment* or therap*)).tw. (99597)
9 (delay* adj2 operation*).tw. (958)
10 (patient* adj2 delay*).tw. (9582)
11 (care adj2 delay*).tw. (1975)
12 (system adj2 delay*).tw. (1135)
13 delay*.kf. (10480)
14 (late adj4 (presentation* or diagnos*)).tw,kf. (13985)
15 (postpone* adj3 (presentation* or diagnos* or colonoscop* or surg* or treatment* or therap* or operation*)).tw,kf. (1469)
16 (defer* adj2 (presentation* or diagnos* or colonoscop* or surg* or treatment* or therap* or operation*)).tw,kf. (2490)
17 ((time or timing or timely or untimely) adj2 (presentation* or diagnos* or colonoscop* or surg* or operation* or treatment* or therap*)).tw,kf. (138746)
18 ((interval or intervals) adj4 (presentation* or diagnos* or colonoscop* or surg* or operation* or treatment* or therap*)).tw,kf. (23812)
19 5 or 6 or 7 or 8 or 9 or 10 or 11 or 12 or 13 or 14 or 15 or 16 or 17 or 18 (1428818)
20 Adult/ (4930581)
21 Young Adult/ (818061)
22 Age Factors/ (446214)
23 (young or younger).tw,kf. (644964)
24 "under the age".tw,kf. (14987)
25 "aged under".tw,kf. (3385)
26 early onset.tw,kf. (36991)
27 20 or 21 or 22 or 23 or 24 or 25 or 26 (5639767)
28 comparative study/ (1857196)
29 Follow-Up Studies/ (636777)
30 chang$.tw. (3047736)
31 evaluat$.tw. (3425310)
32 reviewed.tw. (509355)
33 prospective$.tw. (685031)
34 retrospective$.tw. (718864)
35 baseline.tw. (550273)
36 cohort.tw. (518683)
37 consecutive$.tw. (427381)
38 (compare$ or compara$).tw. (4321963)
39 28 or 29 or 30 or 31 or 32 or 33 or 34 or 35 or 36 or 37 or 38 (10743106)
40 4 and 19 and 27 and 39 (5403)
41 limit 40 to yr="1990 -Current" (4963)
42 limit 41 to (english or french or portuguese or spanish) (4566)

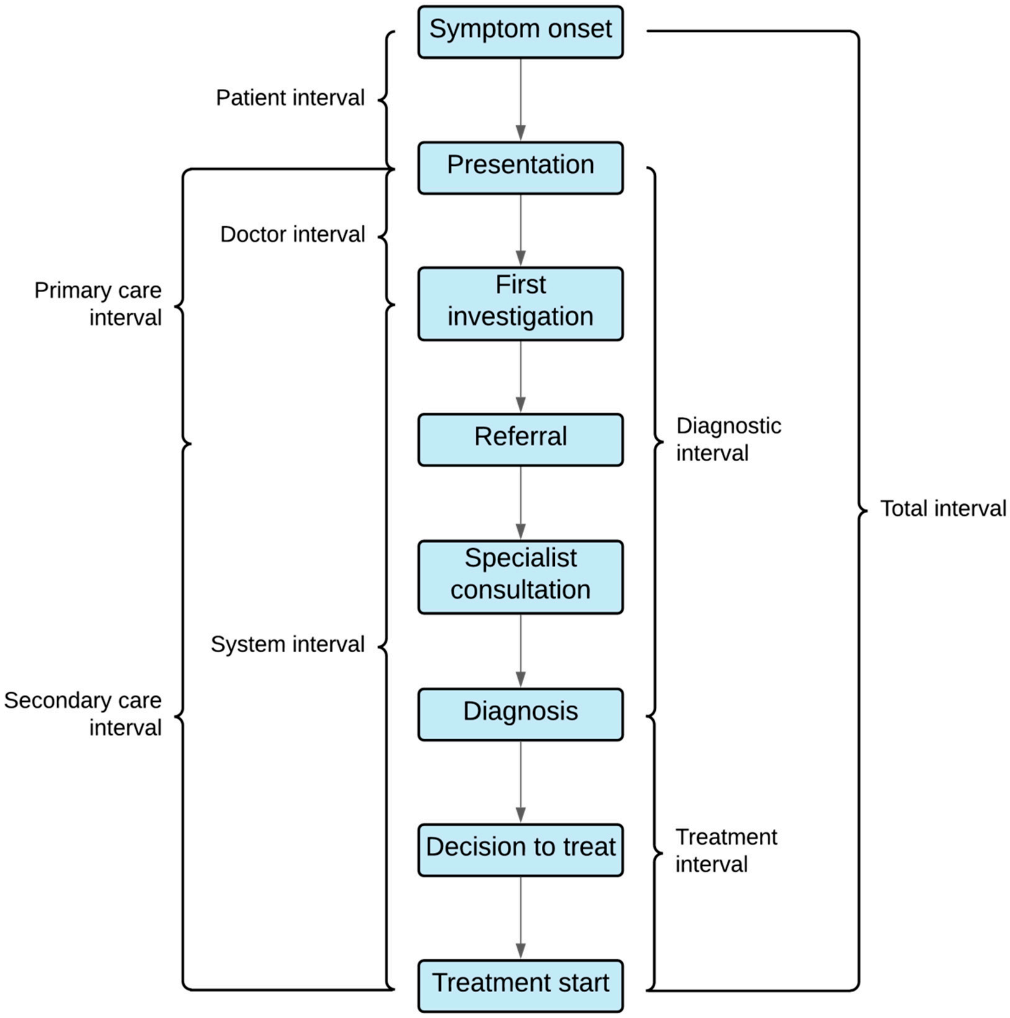

Supplementary Material Figure S3. The pathway to treatment. Time points and delay intervals of interest along the pathway to treatment from symptom onset for patients with colorectal cancer. Intervals are derived from the Aarhus Statement on improving the design and reporting of studies on early cancer diagnosis.[22]

Supplementary Material Table S4A. Scoring for the risk of bias tools, including the Newcastle-Ottawa Scale for Cohort Studies.[23] Blue indicates adherence to a scale item, orange and yellow partial adherence, red non-adherence, and gray unclear adherence.

Study	Selection				Comparability	Outcome		
	**Representativeness of the exposed cohort**	**Selection of the non-exposed cohort**	**Ascertainment of exposure**	**Demonstration that outcome of interest was not present at start of study**	**Comparability of cohorts on the basis of the design or analysis**	**Assessment of outcome**	**Was follow-up long enough for outcomes to occur**	**Adequacy of follow-up of cohorts**
Roder 2019[30]	Somewhat representative of the average delay in the community	Drawn from the same community as the exposed cohort	Secure record (e.g. surgical records)	Yes	Study controls for additional confounding variables	Record linkage	Yes	No statement
Arhi 2019[31]	Truly representative of the average delay in the community	Drawn from the same community as the exposed cohort	Secure record (e.g. surgical records)	Yes	Study controls for additional confounding variables	Record linkage	Yes	No statement
Kaplan 2019[32]	Somewhat representative of the average delay in the community	Drawn from a different source	Secure record (e.g. surgical records)	Yes	Confounding not addressed	Independent blind assessment/medical records	Yes	No statement
Windner 2018[33]	Selected group of users/patients	Drawn from the same community as the exposed cohort	Written self-report	Yes	Confounding not addressed	Self-report	Yes	No statement
Girolamo 2018[13]	Truly representative of the average delay in the community	Drawn from the same community as the exposed cohort	Secure record (e.g. surgical records)	Yes	Confounding not addressed	Record linkage	Yes	More than 10% lost and no description of those lost
Gabriel 2017[34]	Somewhat representative of the average delay in the community	Drawn from the same community as the exposed cohort	Secure record (e.g. surgical records)	Yes	Confounding not addressed	Record linkage	Yes	No statement
Flemming 2017[28]	Somewhat representative of the average delay in the community	Drawn from the same community as the exposed cohort	Secure record (e.g. surgical records)	Yes	Study controls for additional confounding variables	Independent blind assessment/medical records	Yes	Subjects lost to follow-up unlikely to introduce bias (less than 10%) and description of those lost
Sikdar 2017[35]	Somewhat representative of the average delay in the community	Drawn from the same community as the exposed cohort	Secure record (e.g. surgical records)	Yes	Study controls for additional confounding variables	Record linkage	Yes	More than 10% lost and no description of those lost
Chen 2017[36]	Selected group of users/patients	Drawn from the same community as the exposed cohort	Secure record (e.g. surgical records)	Yes	Study controls for additional confounding variables	Independent blind assessment/medical records	Yes	No statement
Kim 2016[12]	Selected group of users/patients	Drawn from the same community as the exposed cohort	Secure record (e.g. surgical records)	Yes	Confounding not addressed	Independent blind assessment/medical records	Yes	No statement
Scott 2016[25]	Selected group of users/patients	Drawn from the same community as the exposed cohort	Secure record (e.g. surgical records)	Yes	Study controls for additional confounding variables	Independent blind assessment/medical records	Yes	No statement
Zhu 2015[29]	Selected group of users/patients	Drawn from the same community as the exposed cohort	Secure record (e.g. surgical records)	Yes	Confounding not addressed	Independent blind assessment/medical records	Yes	No statement
Saluja 2014[40]	Selected group of users/patients	Drawn from the same community as the exposed cohort	Secure record (e.g. surgical records)	Yes	Confounding not addressed	Independent blind assessment/medical records	Yes	No statement
Redaniel 2014[41]	Somewhat representative of the average delay in the community	Drawn from the same community as the exposed cohort	Secure record (e.g. surgical records)	Yes	Study controls for additional confounding variables	Record linkage	Yes	No statement
Gillis 2014[42]	Somewhat representative of the average delay in the community	Drawn from the same community as the exposed cohort	Secure record (e.g. surgical records)	Yes	Study controls for additional confounding variables	Record linkage	No	No statement
de Sousa 2014[43]	Selected group of users/patients	Drawn from the same community as the exposed cohort	Secure record (e.g. surgical records)	Yes	Confounding not addressed	Independent blind assessment/medical records	Yes	No statement

Ben-Ishay 2013[44]	Selected group of users/patients	Drawn from the same community as the exposed cohort	Secure record (e.g. surgical records)	Yes	Confounding not addressed	Independent blind assessment/medical records	Yes	No statement
Esteva 2013[45]	Truly representative of the average delay in the community	Drawn from the same community as the exposed cohort	Secure record (e.g. surgical records)	Yes	Confounding not addressed	Independent blind assessment/medical records	Yes	No statement
Deng 2012[46]	Selected group of users/patients	Drawn from the same community as the exposed cohort	Structured interview	Yes	Confounding not addressed	Self-report	Yes	No statement
Chan 2010[47]	Selected group of users/patients	Drawn from the same community as the exposed cohort	Secure record (e.g. surgical records)	Yes	Confounding not addressed	Independent blind assessment/medical records	Yes	No statement
Tohme 2008[48]	Selected group of users/patients	Drawn from the same community as the exposed cohort	Secure record (e.g. surgical records)	Yes	Confounding not addressed	Independent blind assessment/medical records	Yes	No statement
Johnston 2004[51]	Somewhat representative of the average delay in the community	Drawn from the same community as the exposed cohort	Secure record (e.g. surgical records)	Yes	Study controls for additional confounding variables	Record linkage	Yes	More than 10% lost and no description of those lost
Robertson 2004[52]	Somewhat representative of the average delay in the community	Drawn from the same community as the exposed cohort	Secure record (e.g. surgical records)	Yes	Study controls for additional confounding variables	Record linkage	Yes	No statement
Marble 1992[53]	Selected group of users/patients	Drawn from the same community as the exposed cohort	Secure record (e.g. surgical records)	Yes	Confounding not addressed	Independent blind assessment/medical records	Yes	No statement
Pearson 2019[27]	Somewhat representative of the average delay in the community	Drawn from the same community as the exposed cohort	Secure record (e.g. surgical records)	Yes	Study controls for additional confounding variables	Record linkage	Yes	More than 10% lost and no description of those lost
Wanis 2017[26]	Selected group of users/patients	Drawn from the same community as the exposed cohort	Secure record (e.g. surgical records)	Yes	Confounding not addressed	Independent blind assessment/medical records	Yes	No statement
Jones 2017[37]	Somewhat representative of the average delay in the community	Drawn from the same community as the exposed cohort	Structured interview	Yes	Study controls for additional confounding variables	Self-report	Yes	No statement
Pita-Fernandez 2016[38]	Selected group of users/patients	Drawn from the same community as the exposed cohort	Secure record (e.g. surgical records)	Yes	Confounding not addressed	Independent blind assessment/medical records	Yes	More than 10% lost and no description of those lost
Zhang 2015[39]	Selected group of users/patients	Drawn from the same community as the exposed cohort	Secure record (e.g. surgical records)	Yes	Confounding not addressed	Self-report	Yes	Subjects lost to follow-up unlikely to introduce bias (less than 10%) and description of those lost
Porter 2005[49]	Selected group of users/patients	Drawn from the same community as the exposed cohort	Secure record (e.g. surgical records)	Yes	Study controls for additional confounding variables	Self-report	Yes	More than 10% lost and no description of those lost
Neal 2005[50]	Truly representative of the average delay in the community	Drawn from the same community as the exposed cohort	Written self-report	Yes	Study controls for additional confounding variables	Self-report	Yes	No statement
Da Silva 2020[54]	Selected group of users/patients	Drawn from the same community as the exposed cohort	Secure record (e.g. surgical records)	Yes	Confounding not addressed	Independent blind assessment/medical records	Yes	No statement
Galadima 2021[57]	Somewhat representative of the average delay in the community	Drawn from the same community as the exposed cohort	Secure record (e.g. surgical records)	Yes	Confounding not addressed	Independent blind assessment/medical records	Yes	No statement
Delisle 2020[55]	Truly representative of the average delay in the community	Drawn from the same community as the exposed cohort	Secure record (e.g. surgical records)	Yes	Confounding not addressed	Independent blind assessment/medical records	Yes	No statement
Di Leo 2020[56]	Selected group of users/patients	Drawn from the same community as the exposed cohort	Secure record (e.g. surgical records)	Yes	Study controls for additional confounding variables	Independent blind assessment/medical records	Yes	No statement

Webber 2020[59]	Truly representative of the average delay in the community	Drawn from the same community as the exposed cohort	Secure record (e.g. surgical records)	Yes	Confounding not addressed	Record linkage	Yes	Subjects lost to follow-up unlikely to introduce bias (less than 10%) and description of those lost
Van Erp 2019[58]	Truly representative of the average delay in the community	Drawn from the same community as the exposed cohort	Secure record (e.g. surgical records)	Yes	Confounding not addressed	Independent blind assessment/medical records	Yes	Subjects lost to follow-up unlikely to introduce bias (less than 10%) and description of those lost
Majano 2021[60]	Truly representative of the average delay in the community	Drawn from the same community as the exposed cohort	Secure record (e.g. surgical records)	Yes	Study controls for additional confounding variables	Record linkage	Yes	No statement
Lima 2021[61]	Truly representative of the average delay in the community	Drawn from the same community as the exposed cohort	Secure record (e.g. surgical records)	Yes	Study controls for additional confounding variables	Record linkage	Yes	More than 10% lost and no description of those lost

Supplementary Material Table S4B. Scoring for the risk of bias tools, including the Aarhus checklist.[22] Blue indicates adherence to a checklist item and red non-adherence. Items left blank indicate the checklist item did not apply to the study.

**Aarhus Checklist**	Roder 2019[30]	Arhi 2019[31]	Kaplan 2019[32]	Windner 2018[33]	Girolamo 2018[13]	Gabriel 2017[34]	Flemming 2017[28]	Sikdar 2017[35]	Chen 2017[36]	Kim 2016[12]	Scott 2016[25]	Zhu 2015[29]	Saluja 2014[40]
Definitions of time points and intervals													
For studies requiring the measurement of an interval, are the beginning and end points of this interval clearly defined?	Yes	Yes	Yes	Yes	Yes	Yes	Yes	Yes	Yes	Yes	Yes	Yes	No
For all time points and intervals described, are there precise, transparent and repeatable definitions, and is the complexity of time points such as the date of first symptom and date of first presentation addressed?	No	Yes	No	No	Yes	No	Yes	Yes	No	No	No	No	No
For studies that require an estimate of the date of first symptom:													
Do the researchers refer to a theoretical framework underpinning definition of this time point?				No					No	No	No	No	No
Is there a discussion of the different biases influencing measurement of this time point?				No					Yes	No	Yes	No	No
For studies that require measurement of a date of first presentation to healthcare:													
Do the researchers discuss the complexity of the date of first presentation?		Yes	No					Yes	No		No		No
For studies that require measurement of a date of referral:													
Do the researchers discuss the nature of the referral and provide adequate detail - for example, whether it was for investigation or consultation by a colleague in secondary care?		Yes			Yes						No		
For studies that require measurement of the date of diagnosis:													
Do the researchers use an existing hierarchical rationale for the date of diagnosis measurement?	No	No	No	No		No	No	No	No	No	No	No	
Measurement													
Is the healthcare context in which the study is based fully described?	No	No	No	Yes	Yes	No	Yes	Yes	No	No	No	No	No
Do the questions on time points and/or intervals clearly derive from stated definitions?	No	Yes	No	No	Yes	No	Yes	Yes	Yes	No	No	No	No
Do researchers acknowledge the need for theoretical validation and make reference to the theoretical framework(s) underpinning measurement and analysis of the time points?	No	No	No	No	No	No	Yes	Yes	No	No	No	No	No
For studies using questionnaires and/or interviews with patients and/or health-care providers:													
Has a validated instrument been used?				No									
Have the researchers included a copy of their instrument?				No									
Is there some discussion of how reliability and validity (trustworthiness) has been established?				No									
Do researchers acknowledge the need for theoretical validation and make reference to the theoretical framework(s) underpinning measurement and analysis of the time points?				No									
Is there discussion of the different biases influencing measurement of the time points, such as how and when the question is asked and who is being asked?				No									
Is the timing of the interview in relation to the date of diagnosis provided?				Yes									
Is there any triangulation of self-reported data with other data sources such as case notes?				No									
Is data analysis described in full including how and why data are categorised, how missing and incomplete date are managed, and how outliers at both ends of the spectrum are accounted for?				No									
For studies using primary case-note audit and database analysis:													
Case-note analysis: is there a clear and precise description of how case-note data were used to ascertain time points with an acknowledgement of limitations of such data?	No	No	No				Yes		Yes	No	No	No	No
For database analysis: is there a thorough description of the database chosen including sampling coverage and completeness of information?	No	Yes			Yes	Yes	Yes	Yes			No		

Arhus Checklist cont…	Redaniel 2014[41]	Gillis 2014[42]	de Sousa 2014[43]	Ben-Ishay 2013[44]	Esteva 2013[45]	Deng 2012[46]	Chan 2010[47]	Tohme 2008[48]	Johnston 2004[51]	Pita-Fernandez 2016[38]	Zhang 2015[39]	Porter 2005[49]	Neal 2005[50]
Definitions of time points and intervals													
For studies requiring the measurement of an interval, are the beginning and end points of this interval clearly defined?	Yes	Yes	Yes	No	Yes	Yes	Yes	Yes	Yes	Yes	Yes	Yes	No
For all time points and intervals described, are there precise, transparent and repeatable definitions, and is the complexity of time points such as the date of first symptom and date of first presentation addressed?	Yes	Yes	No	No	Yes	No	No	No	Yes	Yes	Yes	Yes	No
For studies that require an estimate of the date of first symptom:													
Do the researchers refer to a theoretical framework underpinning definition of this time point?			No	No	Yes	No	No	No		No	Yes	No	Yes
Is there a discussion of the different biases influencing measurement of this time point?			No	No	Yes	No	No	No		Yes	Yes	Yes	Yes
For studies that require measurement of a date of first presentation to healthcare:													
Do the researchers discuss the complexity of the date of first presentation?							No				Yes	Yes	
For studies that require measurement of a date of referral:													
Do the researchers discuss the nature of the referral and provide adequate detail - for example, whether it was for investigation or consultation by a colleague in secondary care?								No					No
For studies that require measurement of the date of diagnosis:													
Do the researchers use an existing hierarchical rationale for the date of diagnosis measurement?	Yes	No	No	No	No	No		No	No	No		Yes	No
Measurement													
Is the healthcare context in which the study is based fully described?	No	Yes	No	No	No	Yes	No	No	Yes	No	No	No	No
Do the questions on time points and/or intervals clearly derive from stated definitions?	No	No	No	No	Yes	No	No	No	Yes	No	No	No	Yes
Do researchers acknowledge the need for theoretical validation and make reference to the theoretical framework(s) underpinning measurement and analysis of the time points?	No	No	No	No	Yes	No	No	No	Yes	No	Yes	No	Yes
For studies using questionnaires and/or interviews with patients and/or health-care providers:													
Has a validated instrument been used?					No	No					No	No	Yes
Have the researchers included a copy of their instrument?					Yes	No					No	Yes	No
Is there some discussion of how reliability and validity (trustworthiness) has been established?					Yes	No					No	Yes	No
Do researchers acknowledge the need for theoretical validation and make reference to the theoretical framework(s) underpinning measurement and analysis of the time points?					Yes	No					Yes	No	Yes
Is there discussion of the different biases influencing measurement of the time points, such as how and when the question is asked and who is being asked?					Yes	Yes					Yes	Yes	Yes
Is the timing of the interview in relation to the date of diagnosis provided?					Yes	Yes					Yes	Yes	No
Is there any triangulation of self-reported data with other data sources such as case notes?					Yes	Yes					Yes	Yes	No
Is data analysis described in full including how and why data are categorised, how missing and incomplete date are managed, and how outliers at both ends of the spectrum are accounted for?					Yes	Yes					No	No	No
For studies using primary case-note audit and database analysis:													
Case-note analysis: is there a clear and precise description of how case-note data were used to ascertain time points with an acknowledgement of limitations of such data?			No	No	Yes	No	No	No	Yes	Yes	No	Yes	
For database analysis: is there a thorough description of the database chosen including sampling coverage and completeness of information?	Yes	Yes							Yes				

Aarhus Checklist cont…	Robertson 2004[52]	Marble 1992[53]	Pearson 2019[27]	Wanis 2017[26]	Jones 2017[37]	Da Silva 2020[54]	Galadima 2021[57]	Delisle 2020[55]	Di Leo 2020[56]	Webber 2020[59]	Van Erp 2019[58]	Majano 2021[60]	Lima 2021[61]
Definitions of time points and intervals													
For studies requiring the measurement of an interval, are the beginning and end points of this interval clearly defined?	Yes	Yes	Yes	Yes	Yes	Yes	Yes	Yes	Yes	Yes	Yes	Yes	Yes
For all time points and intervals described, are there precise, transparent and repeatable definitions, and is the complexity of time points such as the date of first symptom and date of first presentation addressed?	Yes	No	Yes	Yes	Yes	No	No	Yes	No	Yes	Yes	Yes	No
For studies that require an estimate of the date of first symptom:													
Do the researchers refer to a theoretical framework underpinning definition of this time point?		No				No			No		Yes	No	
Is there a discussion of the different biases influencing measurement of this time point?		No				No			Yes		Yes	Yes	
For studies that require measurement of a date of first presentation to healthcare:													
Do the researchers discuss the complexity of the date of first presentation?	Yes	No			Yes			Yes		Yes	Yes		
For studies that require measurement of a date of referral:													
Do the researchers discuss the nature of the referral and provide adequate detail - for example, whether it was for investigation or consultation by a colleague in secondary care?			Yes								Yes		
For studies that require measurement of the date of diagnosis:													
Do the researchers use an existing hierarchical rationale for the date of diagnosis measurement?		No	Yes	No		No	No		No	No		Yes	No
Measurement													
Is the healthcare context in which the study is based fully described?	Yes	No	Yes	Yes	Yes	No	Yes	Yes	No	Yes	Yes	Yes	Yes
Do the questions on time points and/or intervals clearly derive from stated definitions?	No	No	Yes	No	No	No	No	Yes	No	Yes	Yes	Yes	Yes
Do researchers acknowledge the need for theoretical validation and make reference to the theoretical framework(s) underpinning measurement and analysis of the time points?	No	No	Yes	No	No	No	No	No	No	Yes	Yes	No	No
For studies using questionnaires and/or interviews with patients and/or health-care providers:													
Has a validated instrument been used?					No								
Have the researchers included a copy of their instrument?					Yes								
Is there some discussion of how reliability and validity (trustworthiness) has been established?					No								
Do researchers acknowledge the need for theoretical validation and make reference to the theoretical framework(s) underpinning measurement and analysis of the time points?					No								
Is there discussion of the different biases influencing measurement of the time points, such as how and when the question is asked and who is being asked?					No								
Is the timing of the interview in relation to the date of diagnosis provided?					Yes								
Is there any triangulation of self-reported data with other data sources such as case notes?					Yes								
Is data analysis described in full including how and why data are categorised, how missing and incomplete date are managed, and how outliers at both ends of the spectrum are accounted for?					No								
For studies using primary case-note audit and database analysis:													
Case-note analysis: is there a clear and precise description of how case-note data were used to ascertain time points with an acknowledgement of limitations of such data?	Yes	No		Yes	No	No	No		No		Yes		
For database analysis: is there a thorough description of the database chosen including sampling coverage and completeness of information?	Yes		Yes				Yes	Yes		Yes	Yes	Yes	Yes

Supplementary Material Table S5. Detailed comparison of delay measures between younger and older adults with colorectal cancer. Red indicates longer delays among younger patients, blue indicates shorter delays among younger patients, and grey indicates no significant difference or mixed findings.

**Study**	**Finding**
Webber 2020	Presentation to diagnosis<br>Age <35 median 111.5 days (90th percentile 365.5)<br>Age 35-44 median 93.5 days (90th percentile 336)<br>Age 45-49 median 73 days (90th percentile 321)<br>Age 50-54 median 78 days (90th percentile 316)<br>Age 55-59 median 83 days (90th percentile 308)<br>Age 60-64 median 79 days (90th percentile 319)<br>Age 65-69 median 79 days (90th percentile 312)<br>Age 70-74 median 84 days (90th percentile 322)<br>Age 75-79 median 88 days (90th percentile 324)<br>Age 80-84 median 92.5 days (90th percentile 336)<br>Age 85-89 median 94 days (90th percentile 334)<br>Age 90+ median 92 days (90th percentile 338), p<0.0001
Delisle 2020	Presentation to treatment, unadjusted<br>Very short (median 5 days)<br>Age <50 18.3%<br>Age 50-65 17.8%<br>Age 66-74 17.7%<br>Age 75+ 23.2%<br>Short (median 28 days)<br>Age <50 24.1%<br>Age 50-65 19.3%<br>Age 66-74 20.4%<br>Age 75+ 21.1%<br>Moderate (median 56 days)<br>Age <50 18.7%<br>Age 50-65 21.0%<br>Age 66-74 20.1%<br>Age 75+ 20.4%<br>Long (median 88 days)<br>Age <50 16.0%<br>Age 50-65 19.7%<br>Age 66-74 21.7%<br>Age 75+ 18.6%<br>Very long (median 157 days)<br>Age <50 22.9%<br>Age 50-65 22.3%<br>Age 66-74 20.1%<br>Age 75+ 16.6%, p<0.0001
Di Leo 2020	Symptoms to diagnosis, unadjusted<br>0-1 month age <50 18.5%, age 50+ 68.9%<br>2-5 months age <50 22.2%, age 50+ 16.7%<br>6-12 months age <50 33.3%, age 50+ 11.1%<br>12+ months age <50 25.9%, age 50+ 3.3%, p<0.0001
Windner 2018	Symptoms to diagnosis ≥6 months, unadjusted<br>Age <50 OR Reference<br>Age 50-59 OR 0.84 (95% CI 0.31-2.26)<br>Age 60+ OR 0.28 (95% CI 0.10-0.80)
Chen 2017	Symptoms to diagnosis<br>Age <50 median 128 days (IQR 60-265)<br>Age ≥ 50 median 79 days (IQR 31-184), p<0.05<br><br>Symptoms to presentation<br>Age <50 median 60 days (IQR 30-180)<br>Age ≥ 50 median 30 days (IQR 7-120), p<0.01<br><br>Presentation to diagnosis<br>Age <50 median 31 days (IQR 10-79)<br>Age ≥ 50 median 22 days (IQR 6-62), p<0.05

Jones 2017	Presentation to treatment ≥60 days, unadjusted<br>Age <50 OR Reference<br>Age 50-64 OR 0.30 (95% CI 0.17-0.54)<br>Age 65+ OR 0.46 (95% CI 0.25-0.85)<br><br>Presentation to treatment ≥90 days, unadjusted<br>Age <50 OR Reference<br>Age 50-64 OR 0.35 (95% CI 0.18-0.67)<br>Age 65+ OR 0.60 (95% CI 0.31-1.17)
Kim 2016	Symptoms to diagnosis<br>Age ≤45 mean 52.9 days<br>Age 56-65 mean 33.3 days, p<0.001<br><br>Symptoms to diagnosis ≥3 months, unadjusted<br>Age ≤45 OR Reference<br>Age 56-65 OR 0.49 (95% CI 0.37-0.64)
Scott 2016	Symptoms to treatment<br>Age <50 median 217 days<br>Age >50 median 58 days, p<0.0001<br><br>Symptoms to presentation<br>Age <50 median 121 days<br>Age >50 median 21 days, p<0.0001<br><br>Presentation to referral<br>Age <50 median 10 days<br>Age >50 median 7 days, p=0.05
Ben-Ishay 2013	Symptoms to diagnosis<br>Age <50 mean 5.3 months<br>Age ≥50 mean 2.4 months, p=0.002
Robertson 2004	Presentation to treatment<br>Age <50 mean 182 days (95% CI 129-258)<br>Age 50-64 mean 120 days (95% CI 100-145)<br>Age 65-74 mean 132 days (95% CI 116-150)<br>Age 75+ mean 169 days (95% CI 139-205), p=0.038
Arhi 2019	Presentation to diagnosis<br>Age <50 median 108 days (IQR 60-225)<br>Age 50-59 median 91.5 days (IQR 54-198)<br>Age 60-69 median 92 days (IQR 54-189), p < 0.05 compared to <50<br>Age 70-79 median 100 days (IQR 55-216.25)<br><br>Referral to diagnosis<br>Age <50 median 59 days (IQR 35-105)<br>Age 50-59 median 46.5 days (IQR 25-85.25)<br>Age 60-69 median 49 days (IQR 29-83)<br>Age 70-79 median 47 days (IQR 28-87.25), p < 0.05 for all compared to <50
	Presentation to referral<br>Age <50 median 27 days (IQR 1-101)<br>Age 50-59 median 21.5 days (IQR 1-104)<br>Age 60-69 median 21 days (IQR 1-91.5)<br>Age 70-79 median 28 days (IQR 3-117.25)
Deng 2012	Symptoms to treatment, colon cancer<br>Age <50 mean 120.3 days (95% CI 79.8-175.9)<br>Age ≥50 mean 74.4 days (95% CI 60.9-90.9), p=0.035
	Symptoms to treatment, rectal cancer<br>Age <50 mean 99.5 days (95% CI 75.1-129.0)<br>Age ≥50 mean 120.3 days (95% CI 102.5-142.6), p=0.241
Tohme 2008	Symptoms to consultation<br>Age <45 mean 29.7 weeks<br>Age >45 mean 18.6 weeks, p=0.01
	Consultation to diagnosis<br>Age <45 mean 3.2 weeks<br>Age >45 mean 1.6 weeks, p>0.05
Marble 1992	Symptoms to presentation<br>Age ≤45 mean 5.5 months<br>Age >40 mean 1.6 months, p=0.001
	Presentation to diagnosis

	Age ≤45 mean <1 week<br>Age >40 mean <1 week, p>0.05
Neal 2005	Specialist consultation to diagnosis<br>Age <25 mean 22 days (SD 40)<br>Age 25-34 mean 20 days (SD 36)<br>Age 35-44 mean 17 days (SD 27)<br>Age 45-54 mean 15 days (SD 27)<br>Age 55-64 mean 12 days (SD 24)<br>Age 65-74 mean 13 days (SD 26)<br>Age 75+ mean 10 days (SD 23), p<0.001
	Symptoms to specialist consultation<br>Age <25 mean 75 days (SD 95)<br>Age 25-34 mean 123 days (SD 185)<br>Age 35-44 mean 135 days (SD 203)<br>Age 45-54 mean 142 days (SD 365)<br>Age 55-64 mean 127 days (SD 497)<br>Age 65-74 mean 112 days (SD 341)<br>Age 75+ mean 102 days (SD 492), p>0.05
	Referral to specialist consultation<br>Age <25 mean 49 days (SD 70)<br>Age 25-34 mean 62 days (SD 71)<br>Age 35-44 mean 51 days (SD 63)<br>Age 45-54 mean 51 days (SD 61)<br>Age 55-64 mean 44 days (SD 56)<br>Age 65-74 mean 41 days (SD 53)<br>Age 75+ mean 37 days (SD 49), p<0.001
	Symptoms to diagnosis<br>Age <25 mean 94 days (SD 86)<br>Age 25-34 mean 148 days (SD 205)<br>Age 35-44 mean 155 days (SD 207)<br>Age 45-54 mean 160 days (SD 369)<br>Age 55-64 mean 137 days (SD 513)<br>Age 65-74 mean 121 days (SD 322)<br>Age 75+ mean 105 days (SD 384), p=0.001
Majano 2021	Symptoms to first investigation, adjusted, colon cancer<br>Age <45 median +71.5 days (95% CI -56.3-199.4)<br>Age 45-54 median +40.5 days (95% CI -9.5-90.6)<br>Age 55-64 Reference<br>Age 65-74 median +23.7 days (95% CI -16.9-64.3)<br>Age 75-84 median +58.7 days (95% CI 13.9-103.5)<br>Age 85+ median +107.0 days (95% CI 32.4-181.6)<br><br>Symptoms to first investigation, adjusted, rectal cancer<br>Age <45 median +32.0 days (95% CI -12.7-76.7)<br>Age 45-54 median +47.5 days (95% CI 8.2-86.8)<br>Age 55-64 Reference<br>Age 65-74 median +32.0 days (95% CI -1.4-65.4)<br>Age 75-84 median +63.5 days (95% CI 12.6-114.4)<br>Age 85+ median +53.0 days (95% CI -20.3-126.3)<br><br>First investigation to diagnosis, adjusted, colon cancer<br>Age <45 median +1.3 days (95% CI -3.2-5.8)<br>Age 45-54 median +1.7 days (95% CI -1.8-5.1)<br>Age 55-64 Reference<br>Age 65-74 median +1.3 days (95% CI -1.5-4.2)<br>Age 75-84 median +1.7 days (95% CI -1.2-4.5)<br>Age 85+ median +3.0 days (95% CI -0.7-6.7)<br><br>First investigation to diagnosis, adjusted, rectal cancer<br>Age <45 median +0.0 days (95% CI -0.5-0.5)<br>Age 45-54 median +0.0 days (95% CI -0.4-0.4)<br>Age 55-64 Reference<br>Age 65-74 median +0.0 days (95% CI -0.4-0.4)<br>Age 75-84 median +0.0 days (95% CI -0.4-0.4)<br>Age 85+ median +0.0 days (95% CI -0.4-0.4)<br><br>Symptoms to diagnosis, adjusted, colon cancer<br>Age <45 median +82.0 days (95% CI -24.5-188.5)

	Age 45-54 median +73.0 days (95% CI 15.2-130.8)<br>Age 55-64 Reference<br>Age 65-74 median +57.5 days (95% CI 13.1-101.9)<br>Age 75-84 median +101.5 days (95% CI 47.5-155.5)<br>Age 85+ median +189.5 days (95% CI 112.9-266.1)<br><br>Symptoms to diagnosis, adjusted, rectal cancer<br>Age <45 median +59.0 days (95% CI -8.5-126.5)<br>Age 45-54 median +60.2 days (95% CI 9.8-110.7)<br>Age 55-64 Reference<br>Age 65-74 median +42.2 days (95% CI -3.8-88.3)<br>Age 75-84 median +87.2 days (95% CI 28.7-145.8)<br>Age 85+ median 127.5 days (95% CI 13.3-241.7)
Galadima 2021	Diagnosis to treatment<br>Age <50 mean 18.62 days (SD 21.22)<br>Age 50+ mean 19.01 (SD 26.13), p=0.7091
Da Silva 2020	Diagnosis to treatment<br>Age <50 mean 4.2 months (SD 4.6)<br>Age 50+ mean 4.6 months (SD 8.4), p>0.05
Van Erp 2019	Presentation to referral<br>Age ≤50 median 34 days (IQR 1-233)<br>Age 51-60 median 3 days (IQR 1-15)<br>Age 61-70 median 14 days (IQR 1-47)<br>Age 71-80 median 6 days (IQR 1-61)<br>Age 81-90 median 8 days (IQR 1-68), p=0.154
Roder 2019	Diagnosis to treatment >30 days among patients who had surgery, adjusted for sex, socioeconomic status, geography, tumor site, stage, grade, and diagnosis year<br>Age <50 OR Reference<br>Age 50-59 OR 1.20 (95% CI 0.70-2.05)<br>Age 60-69 OR 1.26 (95% CI 0.76-2.08)<br>Age 70-79 OR 1.20 (95% CI 0.73-1.95)<br>Age 80+ OR 1.04 (95% CI 0.63-1.72)<br><br>Diagnosis to treatment >60 days, adjusted<br>Age <50 OR Reference<br>Age 50-59 OR 1.00 (95% CI 0.54-2.27)<br>Age 60-69 OR 1.11 (95% CI 0.54-2.27)<br>Age 70-79 OR 1.10 (95% CI 0.55-2.22)<br>Age 80+ OR 1.25 (95% CI 0.61-2.56)
Kaplan 2019	Symptoms to diagnosis<br>Age 10-19 median 3 months (range 0-35)<br>Age 20-25 median 3 months (range 0-48)<br>Age >25 median 4 months (range 0-48), p = 0.710
Sikdar 2017	Presentation to diagnosis<br>Age <50 median 81 days (75th percentile 177)<br>Age 50-59 median 74 days (75th percentile 158)<br>Age 60-69 median 69 days (75th percentile 172)<br>Age 70-79 median 82 days (75th percentile 223)<br>Age 80+ median 105 days (75th percentile 286), p<0.0001
Wanis 2017	Diagnosis to treatment >30 days, unadjusted<br>Age <50 OR Reference<br>Age 50-59 OR 1.14 (95% CI 0.56-2.29)<br>Age 60-69 OR 1.08 (95% CI 0.57-2.04)<br>Age 70-79 OR 1.57 (95% CI 0.84-2.94)<br>Age 80+ OR 1.34 (95% CI 0.71-2.52)
Pita-Fernandez 2016	Symptoms to diagnosis<br>Age <50 median 4.1 months (IQR 2.0-7.9)<br>Age 50-60 median 3.4 months (IQR 1.9-6.5)<br>Age 60-70 median 3.5 months (IQR 1.8-6.7)<br>Age 70-80 median 3.2 months (IQR 1.6-6.3)<br>Age 80+ median 2.7 months (IQR 1.1-5.4), p=0.100
Zhu 2015	Symptoms to diagnosis<br>Age <30 mean 4.6 months<br>Age >30 mean 6.2 months, p=0.691
Saluja 2014	Symptoms to presentation<br>Age <40 median 6 months (range 1-48)<br>Age >40 median 6 months (range 1-36), no p-value

Esteva 2013	Symptoms to diagnosis<br>Age <50 median 171.0 days (IQR 127.2-246.2)<br>Age 50-64 median 163.0 days (IQR 87.5-295.5)<br>Age 65-74 median 137.0 days (IQR 83.0-255.2)<br>Age 75+ median 159.5 days (IQR 84.0-326.2), p=0.34<br><br>Symptoms to treatment<br>Age <50 median 149.0 days (IQR 104.0-214.0)<br>Age 50-64 median 133.0 (IQR 60.5-254.5)<br>Age 65-74 median 112.5 days (IQR 49.0-224.7)<br>Age 75+ median 132.0 days (IQR 62.5-289.5), p=0.20
Chan 2010	Symptoms to presentation<br>Age <40 mean 7.9 months<br>Age >50 mean 6.6 months, p=0.44
Porter 2005	Symptoms to presentation<br>Age <50 median 36 days (IQR 11-79)<br>Age 50-70 median 32 days (IQR 13-69)<br>Age 70+ median 31 days (IQR 9-81), p=0.84<br><br>Presentation to diagnosis<br>Age <50 median 78 days (IQR 36-190)<br>Age 50-70 median 81 days (IQR 41-169)<br>Age 70+ median 113 days (IQR 55-230), p=0.961<br><br>Diagnosis to treatment (surgery)<br>Age <50 median 37 days (IQR 22-55)<br>Age 50-70 median 17 days (IQR 9-39)<br>Age 70+ median 20 days (IQR 10-46), p=0.341
Johnston 2004	Diagnosis to treatment (radiotherapy)<br>Age <40 median 12 weeks (IQR 7-16)<br>Age 40-49 median 16 weeks (IQR 8-19)<br>Age 50-59 median 16 weeks (IQR 8-20)<br>Age 60-69 median 18 weeks (IQR 12-22)<br>Age 70-79 median 16 weeks (IQR 9-21)<br>Age 80+ median 10 weeks (IQR 7-16), no p-value
Girolamo 2018	Referral to specialist consultation > 14 days, unadjusted<br>Age 15-44 OR Reference<br>Age 45-54 OR 1.04 (95% CI 0.74-1.50)<br>Age 55-64 OR 0.97 (95% CI 0.71-1.37)<br>Age 65-74 OR 0.93 (95% CI 0.68-1.30)<br>Age 75+ OR 0.90 (95% CI 0.66-1.27)<br><br>Referral to treatment > 62 days, unadjusted<br>Age 15-44 OR Reference<br>Age 45-54 OR 1.07 (95% CI 0.87-1.30)<br>Age 55-64 OR 1.23 (95% CI 1.02-1.49)<br>Age 65-74 OR 1.46 (95% CI 1.21-1.76)<br>Age 75+ OR 1.71 (95% CI 1.43-2.06)<br><br>Decision to treat to treatment > 31 days, unadjusted<br>Age 15-44 OR Reference<br>Age 45-54 OR 1.27 (95% CI 0.94-1.73)<br>Age 55-64 OR 1.88 (95% CI 1.44-2.49)<br>Age 65-74 OR 2.21 (95% CI 1.71-2.92)<br>Age 75+ OR 2.30 (95% CI 1.78-3.04)
Gabriel 2017	Diagnosis to treatment, colon cancer<br>Age <50 mean 11.18 days (SD 26.73)<br>Age >60 mean 13.18 days (SD 26.15), p<0.001<br><br>Diagnosis to treatment, rectal cancer<br>Age <50 mean 22.02 days (SD 29.22)<br>Age >60 mean 22.48 days (SD 30.75), p<0.001
Lima 2021	Diagnosis to treatment >60 days, colon cancer, adjusted<br>Age <40 OR Reference<br>Age 40-49 OR 1.09 (95% CI 0.86-1.37)<br>Age 50-59 OR 1.32 (95% CI 1.07-1.64)<br>Age 60-69 OR 1.38 (95% CI 1.12-1.70)<br>Age 70-79 OR 1.47 (95% CI 1.18-1.83)<br>Age 80+ OR 1.29 (95% CI 1.00-1.66)

	Diagnosis to treatment >60 days, rectal cancer, adjusted<br>Age <40 OR Reference<br>Age 40-49 OR 1.15 (95% CI 0.95-1.39)<br>Age 50-59 OR 1.36 (95% CI 1.14-1.62)<br>Age 60-69 OR 1.34 (95% CI 1.13-1.60)<br>Age 70-79 OR 1.43 (95% CI 1.20-1.72)<br>Age 80+ OR 1.55 (95% CI 1.26-1.91)
Pearson 2019	Referral to diagnosis<br>Age <25 median 1 day (IQR 0-3)<br>Age 25-44 median 18 days (IQR 2-55)<br>Age 45-49 median 24 days (IQR 8-55.5)<br>Age 50-54 median 24 days (IQR 10-52)<br>Age 55-59 median 25 days (IQR 11-55)<br>Age 60-64 median 25 days (IQR 14-48)<br>Age 65-69 median 25 days (IQR 13-51)<br>Age 70-74 median 26 days (IQR 14-54)<br>Age 75-79 median 27 days (IQR 12-58)<br>Age 80-84 median 27 days (IQR 10-55)<br>Age 85+ median 20 days (IQR 4-47), no p-value<br><br>Referral to diagnosis >25 days, adjusted for sex, ethnicity, socioeconomic status, tumor characteristics, diagnostic route and tests, and co-morbidity<br>Age <25 OR 0.35 (95% CI 0.24-0.52)<br>Age 25-44 OR 0.92 (95% CI 0.81-1.04)<br>Age 45-49 OR 0.95 (95% CI 0.83-1.09)<br>Age 50-54 OR 0.95 (95% CI 0.86-1.05)<br>Age 55-59 OR 0.95 (95% CI 0.84-1.04)<br>Age 60-64 OR Reference<br>Age 65-69 OR 0.96 (95% CI 0.90-1.04)<br>Age 70-74 OR 1.03 (95% CI 0.96-1.11)<br>Age 75-79 OR 1.05 (95% CI 0.98-1.13)<br>Age 80-84 OR 1.08 (95% CI 1.00-1.16)<br>Age 85+ OR 0.92 (95% CI 0.85-0.99)
Flemming 2017	Diagnosis to treatment ≥42 days, adjusted for sex, socioeconomic status, co-morbidity, surgeon volume, geography, cancer stage, and provider type<br>Age 20-59 RR Reference<br>Age 60-59 RR 1.43 (95% CI 1.15-1.77)<br>Age 70-79 RR 1.38 (95% CI 1.12-1.70)<br>Age 80+ RR 1.75 (95% CI 1.41-2.17)<br><br>Diagnosis to treatment >90th percentile, adjusted<br>Age 20-59 RR Reference<br>Age 60-59 RR 1.38 (95% CI 1.01-1.88)<br>Age 70-79 RR 1.26 (95% CI 0.93-1.71)<br>Age 80+ RR 1.77 (95% CI 1.30-2.41)<br><br>Quantile regression reporting difference in days showed similar findings
Zhang 2015	Symptoms to presentation >1 month, unadjusted<br>Age <50 OR Reference<br>Age 50-65 OR 5.97 (95% CI 3.13-12.25)<br>Age 65-80 OR 1.73 (95% CI 0.87-3.68)<br>Age 80-90 OR 6.05 (95% CI 3.09-12.74)
Redaniel 2014	Diagnosis to treatment, adjusted for gender, geography, ethnicity, tumor characteristics, and socioeconomic status<br>Age 15-44 Reference<br>Age 45-54 1.72 additional days (95% CI 0.60-2.85)<br>Age 55-64 2.92 additional days (95% CI 1.76-4.08)<br>Age 65-74 3.76 additional days (95% CI 2.58-4.93)<br>Age 75+ 3.48 additional days (95% CI 2.32-4.63)
Gillis 2014	Specialist appointment to treatment<br>Age <50 median 27 days<br>Age 50-65 median 30 days<br>Age >65 median 32 days, p<0.001<br><br>Specialist appointment to treatment >32 days, adjusted for sex, institution type, co-morbidity, income quintile, geography, hospital volume, year of diagnosis, tumor characteristics, and pre-operative tests/consultation

	Age <50 OR 0.854 (95% CI 0.714-1.021)<br>Age 50-65 OR Reference<br>Age >65 OR 1.117 (95% CI 1.018-1.225)
de Sousa 2014	Symptoms to diagnosis<br>Age <50 mean 6.3 months (SD 4.0)<br>Age ≥50 mean 9.7 months (SD 6.3), p<0.0001

Supplementary Material Table S6. Advanced stage at diagnosis comparing older and younger patients. Red indicates worse outcomes among younger patients, blue indicates better outcomes among younger patients, and grey indicates no significant difference.

Study	Finding
Galadima 2021	Stage III/IV, unadjusted<br>Age <50 OR Reference<br>Age 50+ OR 0.51 (95% CI 0.42-0.61)
Arhi 2019	Stage III/IV, unadjusted<br>Age <50 OR Reference<br>Age 50-59 OR 0.89 (95% CI 0.70-1.12)<br>Age 60-69 OR 0.89 (95% CI 0.72-1.11)<br>Age 70-79 OR 0.69 (95% CI 0.56-0.85)
Kaplan 2019	Duke's Stage C/D, unadjusted<br>Age 10-19 OR Reference<br>Age 20-25 OR 1.03 (95% CI 0.42-2.38)<br>Age >25 OR 0.35 (95% CI 0.15-0.78)
Gabriel 2017	Stage III/IV colon cancer, unadjusted<br>Age <50 OR Reference<br>Age >60 OR 0.52 (95% CI 0.51-0.53)<br><br>Stage III/IV rectal cancer, unadjusted<br>Age <50 OR Reference<br>Age >60 OR 0.61 (95% CI 0.60-0.63)
Chen 2017	Stage III/IV, unadjusted<br>Age <50 OR Reference<br>Age 50+ OR 0.66 (95% CI 0.45-0.97)
Kim 2016	Stage III/IV, unadjusted<br>Age ≤45 OR Reference<br>Age 56-65 OR 0.74 (95% CI 0.62-0.88)
Zhu 2015	Stage III/IV, unadjusted<br>Age <30 OR Reference<br>Age >30 OR 0.31 (95% CI 0.19-0.50)
Marble 1992	Duke's Stage C/D, unadjusted<br>Age ≤40 OR Reference<br>Age >40 OR 0.41 (95% CI 0.18-0.91)
Da Silva 2020	Stage III/IV, unadjusted<br>Age <50 OR Reference<br>Age 50+ OR 0.41 (95% CI 0.16-0.97)
Pita-Fernandez 2016	Stage III/IV colon cancer, adjusted for gender<br>Age increase 1 year OR 0.99 (95% CI 0.97-1.00, p=0.045)<br><br>Stage III/IV rectal cancer, adjusted for gender<br>Age increase 1 year OR 1.00 (95% CI 0.98-1.03, p=0.841)
Scott 2016	Stage III/IV, unadjusted<br>Age <50 OR Reference<br>Age >50 OR 0.61 (95% CI 0.28-1.28)
Saluja 2014	Stage III/IV, unadjusted<br>Age <40 OR Reference<br>Age >40 OR 0.53 (95% CI 0.26-1.04)
de Sousa 2014	Stage III/IV, unadjusted<br>Age <50 OR Reference<br>Age 50+ OR 0.68 (95% CI 0.36-1.28)
Ben-Ishay 2013	Stage III/IV, unadjusted<br>Age <50 OR Reference<br>Age 50+ OR 0.61 (95% CI 0.27-1.33)
Chan 2010	Stage III/IV, unadjusted<br>Age <40 OR Reference<br>Age ≥50 OR 0.69 (95% CI 0.31-1.54)
Tohme 2008	Stage III/IV, unadjusted<br>Age <45 OR Reference<br>Age >45 OR 0.75 (95% CI 0.39-1.44)

Author and Year	Younger adults Advanced stage	Younger adults Early stage	Older adults Advanced stage	Older adults Early stage	Weight	Odds Ratio [95% CI]
Colorectal cancer						
Galadima 2021	358	164	2090	1893	12.94%	1.98 [1.63, 2.40]
Da Silva 2020	24	8	66	54	3.52%	2.45 [1.02, 5.90]
Kaplan 2019	125	41	112	115	8.28%	3.13 [2.02, 4.85]
Arhi 2019	244	160	3088	2572	12.73%	1.27 [1.03, 1.56]
Chen 2017	181	72	145	87	9.30%	1.51 [1.03, 2.21]
Kim 2016	385	308	874	949	13.29%	1.36 [1.14, 1.62]
Zhu 2015	59	22	2229	2669	7.40%	3.21 [1.96, 5.26]
de Sousa 2014	38	22	67	57	5.57%	1.47 [0.78, 2.77]
Saluja 2014	50	16	62	38	4.95%	1.92 [0.96, 3.83]
Chan 2010	27	23	21	26	4.05%	1.45 [0.65, 3.23]
Tohme 2008	24	19	137	144	5.43%	1.33 [0.70, 2.53]
Marble 1992	27	23	16	34	3.94%	2.49 [1.11, 5.63]
Random Effects Model for Subgroup (Q = 32.16, df = 11, p < .01; I² = 68.8%, τ² = 0.07)						1.79 [1.46, 2.21]
Colon cancer						
Ben-Ishay 2013	20	11	104	94	4.14%	1.64 [0.75, 3.61]
Rectal cancer						
Scott 2016	30	26	23	33	4.46%	1.66 [0.78, 3.50]
Random Effects Model for All Studies (Q = 32.17, df = 13, p < .01; I² = 62.5%, τ² = 0.06)					100.00%	1.77 [1.47, 2.14]
Test for Subgroup Differences: QM = 0.06, df = 2, p = 0.97						

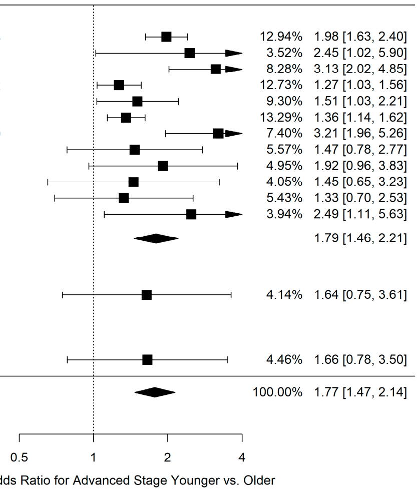

Supplementary Material Figure S7. Random effects meta-analysis of advanced stage at diagnosis by age category. This sensitivity analysis excludes the large Gabriel et al. [34] study. Subgroup analyses were performed by type of cancer studied. Advanced stage was defined as Stage III/IV and early stage was defined as Stage I/II.

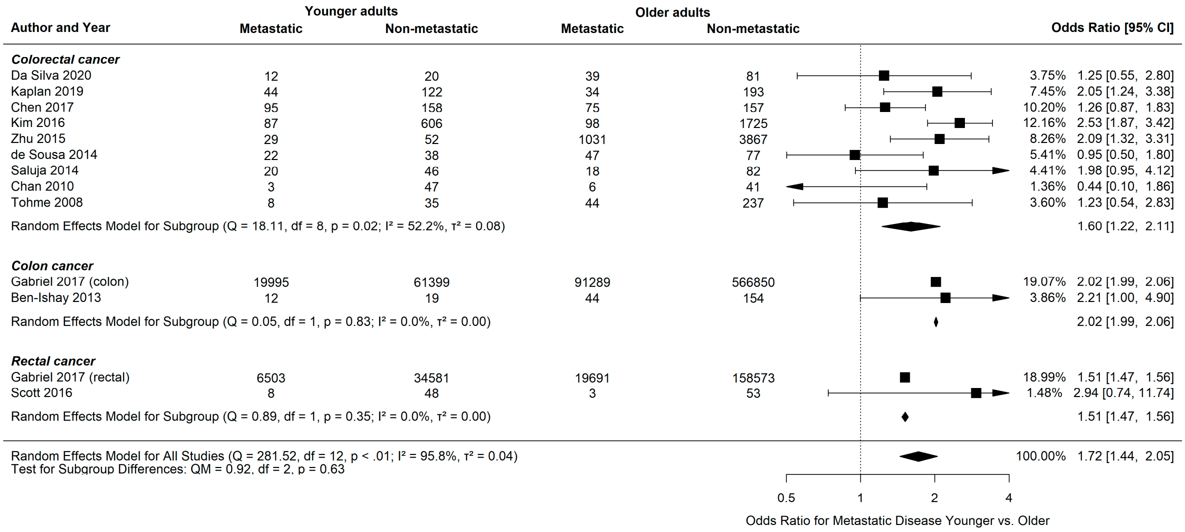

Supplementary Material Figure S8. Random effects meta-analysis of metastatic disease at diagnosis by age category. Subgroup analyses were performed by type of cancer studied.

Supplementary Material Table S9. Survival and recurrence outcomes among younger and older patients. Red indicates worse outcomes among younger patients, blue indicates better outcomes among younger patients, and grey indicates no significant difference.

Study	Finding
**Survival**	
Kim 2016	5-year cancer specific survival<br>Age ≤45 81.2%<br>Age 56-65 87.8%, p<0.001
Marble 1992	5-year overall survival<br>Age ≤40 51%<br>Age >40 75%, p=0.01
Kaplan 2019	Overall survival, median follow-up 33.6 months, unadjusted<br>Age 10-25 OR Reference<br>Age >25 OR 0.61 (95% CI 0.41-0.90)
	Overall survival, median follow-up 33.6 months, adjusted for gender, tumor characteristics, presentation, and stage<br>Age 10-25 OR Reference<br>Age >25 OR 1.05 (95% CI 0.62-1.78)
Da Silva 2020	Overall mortality 31.5%, reports mortality rate similar between age <50 and 50+ (p=0.29)
Scott 2016	5-year overall survival<br>Age <50 64%<br>Age >50 71%, p=0.54
Saluja 2014	Overall survival<br>Age <40 38% at 48 months<br>Age >40 36% at 48 months, p=0.41
de Sousa 2014	Overall survival, mean follow-up 40.8 months (SD 6.4)<br>Age <50 69%<br>Age 50+ 61%, p=0.2482
Ben-Ishay 2013	Overall survival, mean follow-up 3.6 years<br>Age <50 58.1%<br>Age 50+ 61.0%, p=0.92
Tohme 2008	5-year overall survival<br>Age <45 52%<br>Age >45 58.3%, p=0.688<br><br>5-year overall survival, adjusted for sex, family history, symptoms, tumor characteristics, delay measures, and adjuvant therapy<br>Age <45 RR Reference<br>Age >45 RR 0.89 (95% CI 0.20-4.06)
Delisle 2020	Overall survival, adjusted for delay, sex, income, comorbidity, year of diagnosis, distance to referral center, cancer site, grade, and stage<br>Age <50 HR Reference<br>Age 50-65 HR 1.27 (95% CI 1.08-1.49)<br>Age 66-74HR 1.80 (95% CI 1.53-2.12)<br>Age 75+ HR 3.34 (95% CI 2.86-3.91)
Girolamo 2018	1-year overall survival, unadjusted<br>Age 15-44 OR Reference<br>Age 45-54 OR 1.09 (95% CI 0.96-1.25)<br>Age 55-64 OR 1.43 (95% CI 1.27-1.60)<br>Age 65-74 OR 1.77 (95% CI 1.59-1.99)<br>Age 75+ OR 2.62 (95% CI 2.35-2.94)
Gabriel 2017	30-day mortality for colon cancer, unadjusted<br>Age <50 OR Reference<br>Age >60 OR 5.05 (95% CI 4.73-5.41)<br><br>30-day mortality for rectal cancer, unadjusted<br>Age <50 OR Reference<br>Age >60 OR 6.66 (95% CI 5.82-7.66)
Flemming 2017	Overall survival, adjusted for sex, socioeconomic status, co-

	morbidity, tumor characteristics, geography, colonoscopy timing, and surgeon volume<br>Age 20-59 HR Reference<br>Age 60-69 HR 1.16 (95% CI 0.99-1.37)<br>Age 70-79 HR 1.57 (95% CI 1.35-1.82)<br>Age 80+ HR 2.94 (95% CI 2.51-3.43)<br><br>Cancer specific survival, adjusted for sex, socioeconomic status, co-morbidity, tumor characteristics, geography, colonoscopy timing, and surgeon volume<br>Age 20-59 HR Reference<br>Age 60-69 HR 1.07 (95% CI 0.89-1.29)<br>Age 70-79 HR 1.27 (95% CI 1.07-1.51)<br>Age 80+ HR 1.92 (95% CI 1.59-2.33)
Wanis 2017	Overall survival, median follow-up 2.7 year, adjusted for delay measure, tumor characteristics, margin status, and adjuvant treatment<br>Age <50 HR Reference<br>Age 50-59 HR 1.825 (95% CI 0.6633-5.024)<br>Age 60-69 HR 1.175 (95% CI 0.4455-3.102)<br>Age 70-79 HR 2.607 (95% CI 1.037-6.551)<br>Age 80+ HR 3.791 (95% CI 1.503-9.561)
Redaniel 2014	Excess mortality, adjusted for delay measure, geography, ethnicity, socioeconomic status, tumor characteristics, and time period<br>Age 15-44 HR Reference<br>Age 45-54 HR 1.47 (95% CI 1.09-1.97)<br>Age 55-64 HR 1.46 (95% CI 1.11-1.93)<br>Age 65-74 HR 1.74 (95% CI 1.33-2.28)<br>Age 75+ HR 2.71 (95% CI 2.07-3.54)

Recurrence

Kaplan 2019	Event-free survival, median follow-up 33.6 months<br>Age 10-19 median 29.0 months<br>Age 20-25 median 29.9 months<br>Age >25 median 61.6 months, p=0.003
Kim 2016	Recurrence after curative resection, unadjusted<br>Age ≤45 OR Reference<br>Age 56-65 OR 0.74 (95% CI 0.59-0.93)
Da Silva 2020	Any recurrence<br>Age <50 38.1%<br>Age 50+ 34.0%, p=0.5125
de Sousa 2014	Cancer-free survival, mean follow-up 40.8 months (SD 6.4)<br>Age <50 63%<br>Age 50+ 62%, p=0.9218
Tohme 2008	Locoregional recurrence after curative surgery, unadjusted<br>Age <45 14.3%<br>Age >45 8.6%, p=0.3
